# Systemic activation of NRF2 contributes to the therapeutic efficacy of clinically-approved KRAS-G12C anti-cancer drugs

**DOI:** 10.1038/s41416-025-03162-7

**Published:** 2025-09-01

**Authors:** Liam Baird, Lin Zhang, Takanori Hidaka, Lyu Xi, Ke Wang, Keiko Tateno, Tatsuro Iso, Takafumi Suzuki, Kazuki Kumada, Fumiki Katsuoka, Kengo Kinoshita, Masayuki Yamamoto

**Affiliations:** 1 Department of Biochemistry and Molecular Biology, Tohoku University, Tohoku Medical Megabank Organization, Sendai, Japan; 2 Advanced Research Center for Innovations in Next-Generation Medicine (INGEM), Tohoku University, Sendai, Japan; 3 Tohoku Medical Megabank Organization, Tohoku University, Sendai, Miyagi Japan; 4 Department of Applied Information Sciences, Graduate School of Information Sciences, Tohoku University, Sendai, Miyagi Japan; 5 Department of Integrative Genomics, Tohoku University, Tohoku Medical Megabank Organization, Sendai, Japan

**Keywords:** Lung cancer, Targeted therapies

## Abstract

**Background:**

The development and clinical success of KRAS^G12C^ inhibitors was a landmark achievement in anti-cancer drug development, as oncogenic KRAS had long been considered an intractable therapeutic target. Patients with *KRAS* mutant lung cancers frequently present with co-mutations in the KEAP1-NRF2 pathway, and because genetic activation of NRF2 results in resistance to all current anti-cancer therapies, we were motivated to explore how aberrant activation of NRF2 impacts the clinical response to KRAS^G12C^ inhibitors.

**Methods:**

A broad range of techniques, including genetic knockouts, scRNA-seq and surface plasmon resonance, were used to determine the effect of KRAS^G12C^ drugs on NRF2.

**Results:**

At physiologically-relevant concentrations, both of the clinically-approved KRAS^G12C^ inhibitors Sotorasib and Adagrasib also function as inducers of NRF2. Mechanistically, the same cysteine-targeting functionality which allows these electrophilic drugs to inhibit the mutant KRAS^G12C^ protein also facilitates their binding to cysteine-based sensors in KEAP1, resulting in the upregulation of the NRF2-dependent gene expression program.

**Conclusions:**

The activation of NRF2 by KRAS-G12C inhibitors represents a unique example of anti-cancer drugs which positively regulate the activity of a protein which is normally considered to be an oncogene. In both the malignant cells of the tumour and immune cells within the microenvironment, activation of NRF2 by electrophilic KRAS inhibitors positively contributes to the clinical efficacy of these drugs by promoting anti-cancer immunity. This unprecedented situation, in which the NRF2-dependent oxidative stress response is induced globally within cancer patients, has a number of important clinical implications, particularly in relation to ongoing combination chemotherapy clinical trials, as well as for selecting patient populations which may derive the most benefit from G12Ci anti-cancer drugs.

## Introduction

Lung cancer is the leading cause of cancer-related deaths, with genomic studies revealing mutations in *TP53*, *KRAS*, *KEAP1* and *STK11* to be the main genetic drivers of the disease [1, 2]. The oncogene *KRAS* encodes a small GTPase, which functions as a molecular switch by cycling between an inactivate GDP-bound state and active GTP-bound state, and is mutated in 33% of lung adenocarcinoma (LUAD) patients [3]. Activation of KRAS occurs downstream of receptor tyrosine kinase signaling, and promotes cell survival and proliferation which is mediated by upregulation of MAPK signaling [4]. In tumours, constitutive activation of KRAS is achieved through point mutations at codons G12, G13 or Q61, which function to lock KRAS into the active GTP-bound state, and thus confer upon the cancer cells the proliferative and pro-survival effects of KRAS signaling [5]. The lack of allosteric regulatory binding pockets and picomolar affinity for GTP/ GDP made it appear that KRAS was undruggable, until a paradigm shift was made in 2013 in which crystallography analysis revealed the existence of a previously undescribed switch-II pocket which could be targeted by small molecules to lock the KRAS-G12C mutant into the inactive GDP-bound state [6]. Significant advances in the potency and selectivity of the KRAS-G12C inhibitors (G12Ci) produced the compound AMG 510, which under the name Sotorasib became the first clinically-approved KRAS inhibitor [7–12].

Patients with *KRAS* mutant lung cancers frequently present with co-mutations in the KEAP1-NRF2 pathway, which results in the constitutive activation of the transcription factor NRF2 within the tumour cells [13]. NRF2 orchestrates the inducible oxidative stress response, and under normal conditions, is negatively regulated by a KEAP1-dependent E3 ubiquitin ligase which targets NRF2 for proteasome-dependent degradation [14, 15]. Genetic inactivation of *KEAP1*, or gain-of-function mutations in the *NFE2L2* gene which encodes NRF2, produce highly malignant tumours which can evade the anti-cancer immune response, and are resistant to all existing anti-cancer therapies, including immune checkpoint inhibitors [16]. Consequently, patients with genetic activation of NRF2 have a poor prognosis and will often progress to the use of second-line therapies [17–19].

In addition to gaining activating mutations in oncogenes such as *KRAS*, malignant tumours must also develop a microenvironment which inhibits anti-cancer immune surveillance while simultaneously facilitating tumour growth [20]. For example, the chemically stressful nature of the tumour microenvironment, which is characterized by altered nutrient availability, immunosuppressive metabolite production and hypoxia, can facilitate tumour growth by directly inhibiting the anti-cancer immune response [21]. Specifically, competition with tumour cells for micronutrients, persistent exposure to antigens within the tumour, or hydrogen peroxide secretion by tumour resident macrophages, can all induce oxidative stress in cytotoxic CD8+ T cells, which results in T cell dysfunction or programmed cell death [21–23]. Across all T cells, CD8+ effector cells display the greatest sensitivity to oxidative stress [24, 25], and as these cells are primarily responsible for the killing of cancer cells, regulation of the oxidative stress response in CD8+ T cells is critically important for the efficacy of the anti-cancer immune response.

Similarly, the cellular composition and immune infiltration profile of the tumour is critically important for malignant cancer development. In this regard, the recruitment of myeloid lineage cells, which include monocytes, tumour associated macrophages (TAMs) and myeloid-derived suppressor cells (MDSCs), contribute to the generation of an immunosuppressive microenvironment through the production of cytokines including IL-10 and TGFβ, which function within the tumour niche to inhibit the anti-cancer activities of cytotoxic T cells [26–28]. Furthermore, tumour infiltrating myeloid cells also express ligands which can activate the PD-1, LAG-3 and TIGIT checkpoints in T cells, in order to further repress anti-cancer immunity [29]. Thus, as myeloid cells are generally characterized as being tumour promoting, their infiltration into tumours is associated with therapy resistance and a poor prognosis for patients [28]. In light of these facts, therapeutic approaches which aim to inhibit the immunosuppressive function of myeloid cells, which include repolarization away from suppressive M2-type states, are being actively developed in order to complement and enhance the efficacy of existing immune checkpoint inhibitor drugs [30, 31].

As genetic activation of NRF2 results in resistance to all current anti-cancer therapies, we were interested in investigating how aberrant activation of NRF2 may impact the clinical response to the new class of KRAS^G12C^ inhibitor drugs. In this study, we found that at clinically-relevant doses, the KRAS^G12C^-inhibitors Sotorasib and Adagrasib function as NRF2 inducers by utilizing a mechanism which is mediated by KEAP1 inactivation, and which is completely independent of *KRAS*^*G12C*^ mutant status or KRAS inhibition. In malignant cells, NRF2 activation by G12Ci drugs contributes to their anti-cancer activity by promoting immune surveillance, while in immune cells, NRF2 activation promotes anti-cancer immunity by reducing oxidative stress, and repolarizing myeloid cells towards the anti-cancer M1 lineage. Thus, through the promotion of anti-cancer immunity through multiple parallel pathways, activation of NRF2 by electrophilic KRAS^G12C^-inhibitors positively contributes to the clinical efficacy of these anti-cancer drugs.

## Results

### The KRAS G12C inhibitor Sotorasib activates NRF2

**Fig. 1:**
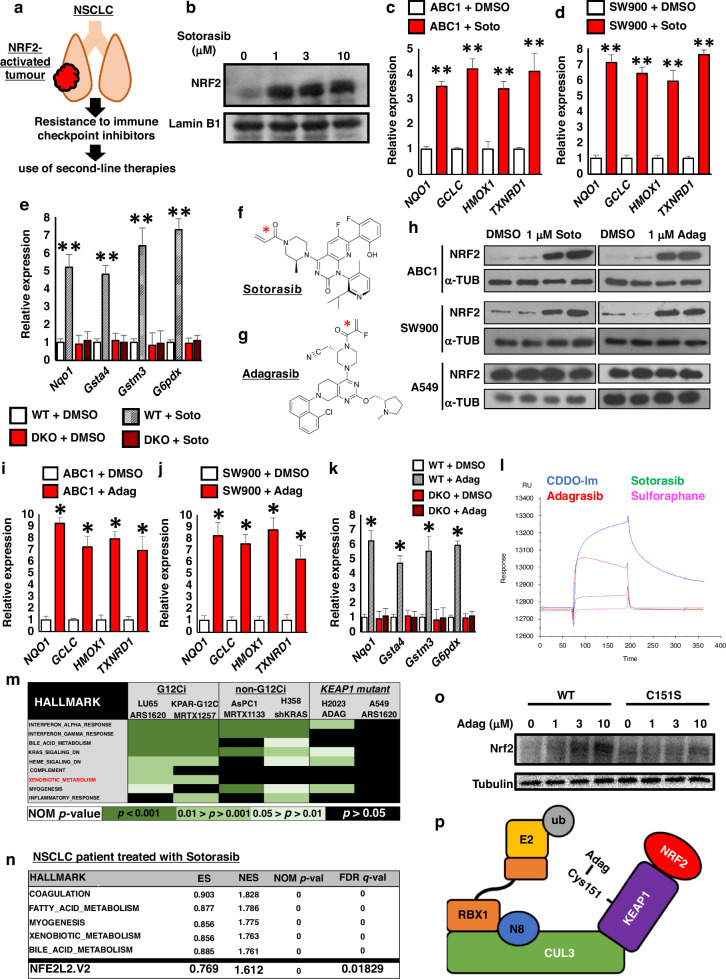
KRAS G12Ci drugs are classical electrophile-based NRF2 inducers. **a** As genetic activation of NRF2 in NSCLC results in resistance to all existing therapies, these tumours will be enriched in patient populations receiving second-line therapies, which includes novel KRAS-G12C drugs. **b** Immunoblot showing significant stabilization of the NRF2 protein in ABC1 cells treated with the indicated concentrations of Sotorasib for 5 h. **c**, **d** The relative expression of representative classical NRF2 target genes in NSCLC cells treated with 1 μM Sotorasib (Soto) for 5 hrs, determined using RT-qPCR. Each experiment was performed at least 3 times. Data represent mean ± SD, *N* = 6. **e** The relative expression of representative classical NRF2 target genes in isogenic WT and Keap1-Nrf2 DKO Hepa1 cells after treatment 1 μM Sotorasib (Soto) for 5 hrs, determined using RT-qPCR. Each experiment was performed at least 3 times. Data represent mean ± SD, *N* = 6. **f**, **g** Chemical structures of Sotorasib and Adagrasib. The cysteine-targeting electrophilic warheads are highlighted with a red star. **h** Immunoblot showing significant stabilization of the NRF2 protein in response to 1 μM Sotorasib (Soto) or Adagrasib (Adag) in ABC1 and SW900 cells, which both have a functional KEAP1-NRF2 pathway, but not in A549 cells, which have constitutive genetic activation of NRF2. **i**, **j** The relative expression of representative classical NRF2 target genes in ABC1 (**i**) and SW900 (**j**) cells, which both have a functional KEAP1-NRF2 pathway, treated with 1 μM Adagrasib (Adag) for 5 hrs, determined using RT-qPCR. Each experiment was performed at least 3 times. Data represent mean ± SD, *N* = 6. **k** The relative expression of representative classical NRF2 target genes in isogenic WT and Keap1-Nrf2 DKO Hepa1 cells after treatment 1 μM Adagrasib (Adag) for 5 hrs, determined using RT-qPCR. Each experiment was performed at least 3 times. Data represent mean ± SD, *N* = 6. **l** A representative surface plasmon resonance sensorgram showing direct binding of both Sotorasib and Adagrasib to the KEAP1 protein. **m** Combined gene set enrichment analysis from six KRAS inhibitor studies shows that the “Xenobiotic_metabolism” hallmark, which is regulated by NRF2, is only upregulated in response to electrophile-based G12Ci compounds, and not by non-G12Ci methods, or in cells with KEAP1 mutations which already display constitutive NRF2 activation. Data from GSE103021, GSE199582, GSE201412 and GSE224439. **n** Gene set enrichment analysis from a human NSCLC patient being treated with Sotorasib shows that both “Xenobiotic metabolism” and “NFE2L2.V2” gene sets are upregulated in human cancer patients. Data were sourced from Tsai et al. 2022. **o** Immunoblot showing significant stabilization of the NRF2 protein in wild type MEFs (WT), but not Keap1-C151S mutant MEFs (C151S), in response to the indicated concentrations of Adagrasib (Adag). **p** Model showing that electrophilic KRAS-G12C inhibitor drugs are able to activate NRF2 by directly binding to cysteine-based sensors in KEAP1, which results in the inhibition of KEAP1’s E3 ubiquitin ligase activity, and thus the stabilization of NRF2. *p < 0.01, **p < 0.001, ns not significant.

Tumours with genetic activation of NRF2 are resistant to all current anti-cancer therapies, including ICIs, and therefore most lung cancer patients with mutations in *KEAP1*, or the NRF2 encoding *NFE2L2*, will progress to second-line treatment strategies [32]. In this regard, Sotorasib is a recently clinically approved second-line NSCLC therapeutic which targets oncogenic *KRAS*^*G12C*^ mutations [9]. As Sotorasib is approved for use in patients who became resistant to first line therapies, a patient population which will be enriched with *KEAP1* and *NFE2L2* mutant tumours, we were interested in understanding how Sotorasib may interact with KEAP1-NRF2 signaling (Fig. 1a) Through this approach, we found that electrophilic KRAS^G12C^ inhibitors function as classical inducers of the NRF2-dependent antioxidant and xenobiotic responses.

In light of the fact that NRF2 is regulated at the level of protein stabilization, in order to gain the most basic insight into a potential role of Sotorasib in NRF2 function, we treated lung cancer cells with a functioning KEAP1-NRF2 system with Sotorasib, and performed an immunoblot for NRF2 stabilisation (Fig. 1b). To our surprise, Sotorasib treatment led to robust stabilization of the NRF2 protein, which encouraged us to further explore the interaction between NRF2 activation and Sotorasib treatment.

As a transcription factor, activation of NRF2 results in the upregulation of a broad range of antioxidant and xenobiotic metabolism pathway genes [33]. Treatment of ABC1 and SW900 lung cancer cells, which both contain a functional KEAP1-NRF2 response, resulted in significant induction of NRF2 target gene expression, including *NQO1*, *GCLC*, *HMOX1* and *TXNRD1* (Fig. 1c, d). Importantly, this induction was not observed in *KEAP1* mutant H2023 or A549 cells (FIG S1A, B), which already exhibit constitutive NRF2 activation, and thus suggests that Sotorasib requires a functional KEAP1 protein to activate NRF2-dependent xenobiotic metabolism gene transcription. Of note, as both ABC1 and SW900 cells do not contain a *KRAS*^*G12C*^ mutation, these results imply that NRF2 activation in response to treatment with Sotorasib occurs independently of KRAS inhibition.

To confirm the requirement of a functional KEAP1-NRF2 axis for the induction of NRF2 target genes in response to Sotorasib treatment, we utilized an isogenic cell line pair consisting of WT and Keap1-Nrf2 double knockout (DKO) cells [34]. While NRF2 target genes were induced by Sotorasib in the WT cells, induction was not observed in the DKO cells (Fig. 1e), which supports a model in which Sotorasib requires a functional KEAP1-NRF2 axis in order to induce xenobiotic gene expression.

Sotorasib is able to specifically target the G12C residue in mutant KRAS due to the cysteine-reactive electrophile warhead present within the structure of the drug (Fig. 1f) [9]. As NRF2 is commonly activated by electrophilic compounds that are structurally similar to Sotorasib [35, 36], and which function as NRF2 inducers through direct binding to reactive cysteine residues within KEAP1, we hypothesized that Sotorasib may activate NRF2 by functioning as a classical KEAP1 cysteine-reactive NRF2 inducer.

To test this idea, we treated NSCLC cells with a second clinically-approved G12Ci, Adagrasib, which is structurally unrelated to Sotorasib, but still inactivates KRAS^G12C^ through use of an electrophilic warhead (Fig. 1g) [37, 38]. Consistent with our model, Adagrasib treatment increased NRF2 protein levels and significantly increased the expression of classical NRF2 xenobiotic metabolism target genes *NQO1*, *GCLC*, *HMOX1* and *TXNRD1* in both ABC1 and SW900 cells, but not in *KEAP1* mutant A549 cells (Fig. 1h–j, S1C, D). Furthermore, Adagrasib also increased NRF2 target gene expression in WT, but not Keap1-Nrf2 DKO cells, suggesting that the xenobiotic metabolism gene induction is dependent on a functional KEAP1-NRF2 axis (Fig. 1k). As a third electrophile-based G12Ci compound, ARS-1620 also showed the same NRF2-dependent induction of xenobiotic metabolism genes (FIG S2A–F), the combined data from three different G12Ci compounds all support a model in which these drugs are able to induce NRF2 activation independently of KRAS inhibition, and through a mechanism which is dependent on the presence of an electrophilic warhead within the structure of the drug.

### KRAS G12Ci drugs are classical electrophile-based NRF2 inducers

To confirm that the activation of NRF2 by Adagrasib and Sotorasib is mediated by direct binding to KEAP1, we investigated the binding dynamics of both G12Ci compounds to KEAP1 in a cell-free system using surface plasmon resonance (SPR)(Fig. 1l, S3). Analysis of the resulting sensorgram clearly shows that both Adagrasib and Sotorasib are able to covalently bind to KEAP1, with Adagrasib demonstrating a higher affinity than Sotorasib. As control compounds, we also analyzed the highly potent NRF2 inducer CDDO-Im, and the less potent inducer sulforaphane, which binds to KEAP1 reversibly [39]. Of note, the sensorgram data are consistent with the relative potency of the compounds to induce NRF2 stabilization in cells, with CDDO-Im demonstrating the highest affinity for KEAP1, and the G12Ci compounds both exhibiting greater affinity for KEAP1 than the reversible binding sulforaphane. Together, these data conclusively show that the electrophilic G12Ci drugs are able to directly bind to KEAP1.

As in the clinic, G12Ci drugs will only be administered to patients who have a *KRAS*^*G12C*^ mutation, we wanted to confirm that NRF2 activation also occurs in this genetic context. To this end, we analyzed six published RNA-Seq datasets which were generated under a diverse set of experimental conditions (Fig. 1m, FIG S4A–D). This analysis revealed that, while the “Xenobiotic metabolism” hallmark gene set was enriched in *KRAS*^*G12C*^ mutant LU65 and KPAR cells treated with ARS-1620 and the Adagrasib analogue MRTX1257 respectively, xenobiotic metabolism genes were not upregulated by the KRAS^G12D^ inhibitor MRTX1133, genetic knockdown of KRAS using shKRAS, or using of G12Ci drugs in KEAP1 mutant A549 or H2030 cells, the latter of which contains a *KRAS*^*G12C*^ mutation [8, 40–42]. Taken together, these data clearly demonstrate that NRF2 target genes are specifically induced by electrophile-based G12Ci, and not by other forms of KRAS inhibition, and that this NRF2 induction only occurs in cells with a functional KEAP1-NRF2 axis.

To confirm that G12Ci also induce NRF2 activity in human cancer patients, we analysed gene expression data from a patient undergoing Sotorasib treatment for NSCLC [43]. This analysis revealed that both the “Xenobiotic metabolism” and “NFE2L2.V2” gene signatures are significantly upregulated upon G12Ci treatment (Fig. 1n), and thus confirms that activation of NRF2 is physiologically relevant and occurs in human cancer patients.

Electrophile inducers of NRF2 function by directly binding to sensor cysteine residues in KEAP1, of which Cys151, -273 and -288 are the best characterized examples [44, 45]. Sensor activation leads to the inhibition of KEAP1-dependent ubiquitination of NRF2, which results in the stabilization of the NRF2 protein, and the concomitant upregulation of xenobiotic metabolism genes [15]. While previously published mass spectrometry data of cells treated with Sotorasib suggest that it is able to directly bind to the Cys288 sensor within KEAP1 [9] (FIG S5A), the identity of the sensor responsible for Adagrasib binding is completely unknown. To identify the specific sensor, we utilized genetic knock-in sensor mutants expressed in mouse embryonic fibroblasts (MEFs) [44]. Through this approach, we found that, while WT MEFs are able to respond to Adagrasib treatment by stabilizing Nrf2, this effect was completely lost in C151S mutants, which supports a model in which Cys151 is the KEAP1 sensor responsible for NRF2 activation (Fig. 1o).

In summary, as none of the cell lines used in any of our analyses contain a *KRAS*^*G12C*^ mutation, together our results suggest the existence of a novel secondary mechanism of action of Sotorasib and Adagrasib, which is independent of KRAS inhibition, and is mediated by direct binding to KEAP1 (Fig. 1p). This parallel function of G12Ci drugs results in the stabilization of NRF2, and the concomitant activation of the xenobiotic metabolism and anti-oxidative stress transcriptional programs.

### KRAS G12Ci induce the NRF2-dependent NISP to promote anti-cancer immunity

While G12Ci drugs are potent KRAS inhibitors at a concentration of approximately 100 nM, the plasma concentration achieved in patients at doses optimized for clinical efficacy is above 10 μM [9, 46]. This apparent 100-fold difference in the concentration required to inhibit KRAS and elicit an efficacious anti-cancer response motivated us to explore the possibility that activation of NRF2 may form an important auxiliary component of the clinical effect of G12Ci drugs in patients.

In addition to repressing oncogenic KRAS signaling, the therapeutic efficacy of G12Ci is also mediated through the upregulation of the anti-cancer immune response [9, 40, 47, 48]. Consistent with this fact, in patients, the highest overall survival rate upon G12Ci treatment is observed in PD-L1 negative tumours, which highlights the clinical importance of the anti-cancer immune response for the efficacy of these drugs [49]. Mechanistically, G12Ci treatment promotes tumour immune surveillance through the upregulation of cytokines and chemokines which function in concert to promote anti-tumour immunity [9]. As we recently found that NRF2 promotes the transcriptional upregulation of a similar set of cytokines and chemokines, which we collectively named the NISP [50], we hypothesized that activation of NRF2 by G12Ci may form an integral component of the mechanism through which these drugs promote anti-cancer immunity (FIG. S5B).

**Fig. 2:**
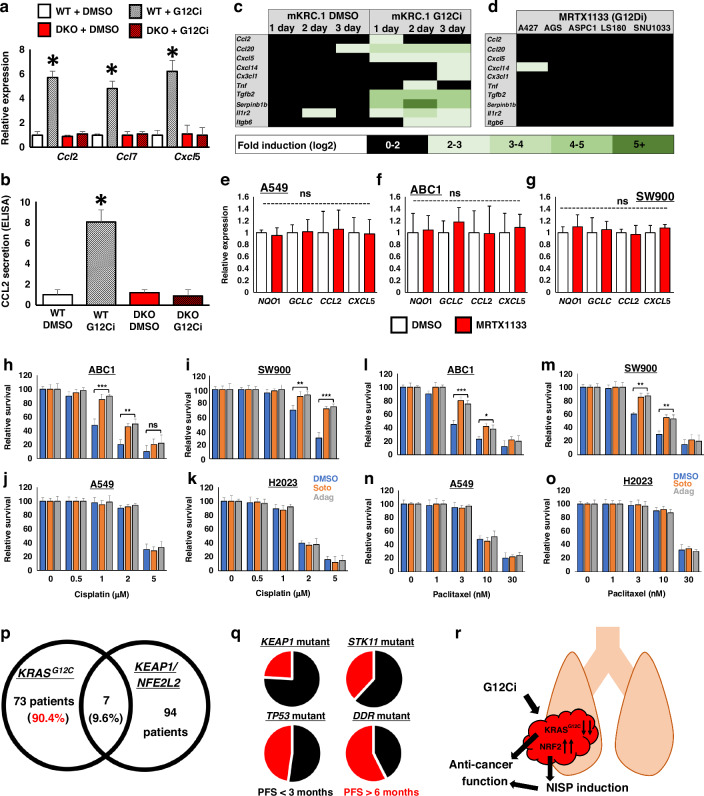
KRAS G12Ci induce the NRF2-dependent NISP to promote anti-cancer immunity. **a** The relative expression of NISP genes in isogenic WT and Keap1-Nrf2 DKO Hepa1 cells after treatment with 1 μM Adagrasib (G12Ci) for 48 hrs, determined using RT-qPCR. Each experiment was performed at least 3 times. Data represent mean ± SD, *N* = 6. **b** ELISA for secreted CCL2 protein, using conditioned media from WT and Keap1-Nrf2 DKO Hepa1 cells, after treatment with 1 μM Adagrasib (G12Ci) for 48 h. Each experiment was performed at least 3 times. Data represent mean ± SD, *N* = 6. **c**, **d** NRF2-induced secretory phenotype (NISP) genes are only induced by electrophilic KRAS-G12C inhibitors (**c**) and not by non-electrophilic KRAS inhibitors (**d**). mKRC.1 and A427 cells are from lung cancers, AGS is from gastric cancer, ASPC1 is from pancreatic cancer, and LS180 and SNU1033 are derived from colon cancers. Data were sourced from Sisler et al. 2023 and Hallin et al. 2022. **e**–**g** The expression of the representative anti-oxidative stress genes NQO1 and GCLC or representative NISP chemokines CCL2 and CXCL5 are not changed by treatment with the non-electrophilic KRAS inhibitor MRTX1133. **h**–**o** Pre-treatment with Sotorasib (orange) or Adagrasib (grey) increases cellular survival in response to the chemotherapeutic drugs cisplatin and paclitaxel in ABC1 and SW900 cells, which both have a functional KEAP1-NRF2 pathway, but not in A549 or H2023 cells, which have constitutive genetic activation of NRF2. **p** The vast majority of human NSCLC patients with KRAS-G12C mutations do not have mutations in either KEAP1 or NRF2, and therefore in most patients, G12Ci treatment will lead to NRF2 induction in the tumour cells. Data were sourced from Clinical lung cancer genome project (CLCGP) 2013. **q** Within human lung cancer patients who received KRAS-G12C inhibitor drugs, those patients whose tumours also had mutations in *KEAP1* had extremely poor progression free survival (PFS) compared to other common mutations like *STK11* and *TP53*, while co-mutations in DNA damage response (DDR) pathway genes had the longest PFS. Data sourced from Negrao et al. 2023. **r** The anti-cancer effect of electrophilic KRAS-G12C inhibitor drugs is mediated by two parallel pathways, namely KRAS inhibition and NRF2 induction. *p < 0.01, **p < 0.005, ***p < 0.001.

To test this model that NRF2 plays an important role in the promotion of G12Ci-induced anti-cancer immunity, we treated WT and Keap1-Nrf2 DKO cells with G12Ci and measured NISP chemokine gene induction in cells without a KRAS^G12C^ mutation. This analysis revealed that, while treatment of WT cells with G12Ci could induce expression of the NISP genes *Ccl2*, *Ccl7* and *Cxcl5*, this gene induction was abolished in the absence of a functional KEAP1-NRF2 axis (Fig. 2a). Consistent with these data, ELISA assays showed that WT, but not Nrf2 deficient cells, were able to secrete CCL2 protein in response to G12Ci treatment (Fig. 2b). Importantly, as these cell lines do not express KRAS^G12C^, these results show that part of the chemokine-induced anti-cancer immune response which is mediated by G12Ci drugs can be modulated independently of KRAS inactivation.

To confirm that NISP gene expression is induced specifically by electrophile-based KRAS inhibitors, and not generally by KRAS inhibition, we compared NISP gene expression in response to G12Ci and the non-cysteine targeting G12Di MRTX1133 [41, 51]. While a broad range of NISP genes were induced by the electrophile-based G12Ci (Fig. 2c), across a panel of five cell lines, NISP gene expression was not induced by G12Di (Fig. 2d). Similarly, in lung cancer cell lines, MRTX1133 was unable to induce xenobiotic metabolism or cytokine gene expression, regardless of *KEAP1* mutation status (Fig. 2e–g). While the transcriptional regulation of cytokine gene expression in KRAS mutant cancers is complex [52], together our data suggest that in certain genetic contexts, electrophile-based NRF2 induction, and not KRAS inhibition per se, is specifically required for NISP gene induction.

In cancer cells, activation of the NRF2-dependent xenobiotic metabolism pathway results in the resistance to chemotherapy drugs [53, 54]. To support the idea that the induction of NRF2 by G12Ci drugs is functionally important, we co-treated lung cancer cells with Sotorasib or Adagrasib along with the anti-cancer drugs cisplatin or paclitaxel (Fig. 2h–o). Consistent with our thesis, G12Ci treatment decreased sensitivity to cisplatin and paclitaxel in *KEAP1* WT cells (ABC1, SW900), but not *KEAP1* mutant (A549, H2023) cells. These data argue that activation of NRF2 in cancer patients receiving either Sotorasib or Adagrasib will be functionally important.

As genetic activation of NRF2 occurs in approximately 25% of NSCLC patients [13], G12Ci-dependent NRF2 induction in tumour cells will be dependent on the mutation profile of the tumour. Specifically, as cancers with pre-existing *KEAP1* or *NFE2L2* mutations exhibit constitutive NRF2 activation, they will not benefit from NRF2 induction by G12Ci drugs, while in contrast, tumours with wild-type KEAP1-NRF2 will benefit from G12Ci-induced NRF2 activation. Thus, to determine the overlap of patients with *KRAS*^*G12C*^ and activating mutations in the KEAP1-NRF2 pathway, we analyzed the genome sequencing data within the Clinical Lung Cancer Genome Project [55]. This large cohort study contains mutational data for *KRAS*, *KEAP1* and *NFE2L2* from 853 lung cancer patients. Analysis of these data revealed that 90.4% of patients with *KRAS*^*G12C*^ mutations have a normally functioning KEAP1-NRF2 pathway, and thus will gain NRF2 activation within the tumour cells upon treatment with G12Ci drugs (Fig. 2p). These clinical data highlight the fact that, in the vast majority of lung cancer patients who will receive G12Ci treatment, these therapies will also activate NRF2 within the tumour cells.

As our data suggest that activation of NRF2 forms an important component of the G12Ci-induced anti-cancer effect, we would expect that tumours with genetic activation of NRF2, which would not benefit from G12Ci-mediated NRF2 activation, would have worse prognosis that tumours with a fully functioning NRF2 response. In good agreement with this model, clinico-genomic analysis of 424 G12Ci-treated NSCLC patients revealed that patients with inactivating mutations in *KEAP1* exhibited a poor response to G12Ci treatment, characterized by early disease progression (Fig. 2q) [56]. Indeed, amongst the most frequently mutated genetic drivers of NSCLC, namely *KEAP1*, *STK11* and *TP53*, *KEAP1* mutations are associated with the worst patient survival in two independent patient cohorts [49, 56]. These real-world data suggest that activation of NRF2 may form a clinically-important component of the G12Ci-dependent anti-cancer effect.

Importantly, in vitro, isogenic loss of *KEAP1* does not result in decreased sensitivity to G12Ci [57], which suggests that the resistance observed in patients is not cell intrinsic, and thus may be mediated by a change in the tumour microenvironment which is absent in the cell culture setting. Taken together, we propose that, in addition to inhibiting mutant KRAS, the anti-cancer effect of G12Ci drugs is also mediated by the electrophile-mediated induction of NRF2, which results in induction of NISP expression, and the concomitant promotion of the anti-cancer immune response (Fig. 2r).

### NRF2 induction broadly activates the anti-oxidative stress response in immune cells

In addition to the malignantly transformed cells, the cellular composition and metabolic profile of the tumour microenvironment plays a critical role in tumour development, progression and response to therapies [20]. Many tumours develop in an environment characterized by extensive oxidative stress, as this can promote tumour formation through a myriad of pathways, including the repression of the anti-cancer immune response [58]. As electrophilic G12Ci drugs are administered orally, clinical treatment will result in systemic NRF2 activation throughout the body of the patient, including within cells of the immune system and the stromal compartments of the tumour.

Due to the critical role that the immune system plays in the anti-cancer response [20], coupled with the fact that oxidative stress within the tumour microenvironment broadly inhibits immune cell effector functions [24, 25], we hypothesized that drug-induced NRF2 activation may positively contribute to anti-cancer immune surveillance by alleviating oxidative stress. As such, NRF2 may represent a novel immunotherapeutic target which would complement existing checkpoint inhibitor strategies. Thus, in order to determine the specific role of drug-induced NRF2 activation in modulating immune cell functions, we carried out scRNA-Seq using human peripheral blood mononuclear cells (PBMCs). NRF2 activation was achieved through treatment with the highly potent triterpenoid CDDO-2P-Im, which exclusively activates NRF2 at low nanomolar concentrations [59, 60]. This allowed us to specifically isolate the NRF2-dependent effects on immune cell gene expression, as G12Ci can also modify other proteins, including the transcription regulator SETD1B which may obscure the NRF2-dependent changes in gene expression [61, 62].

**Fig. 3:**
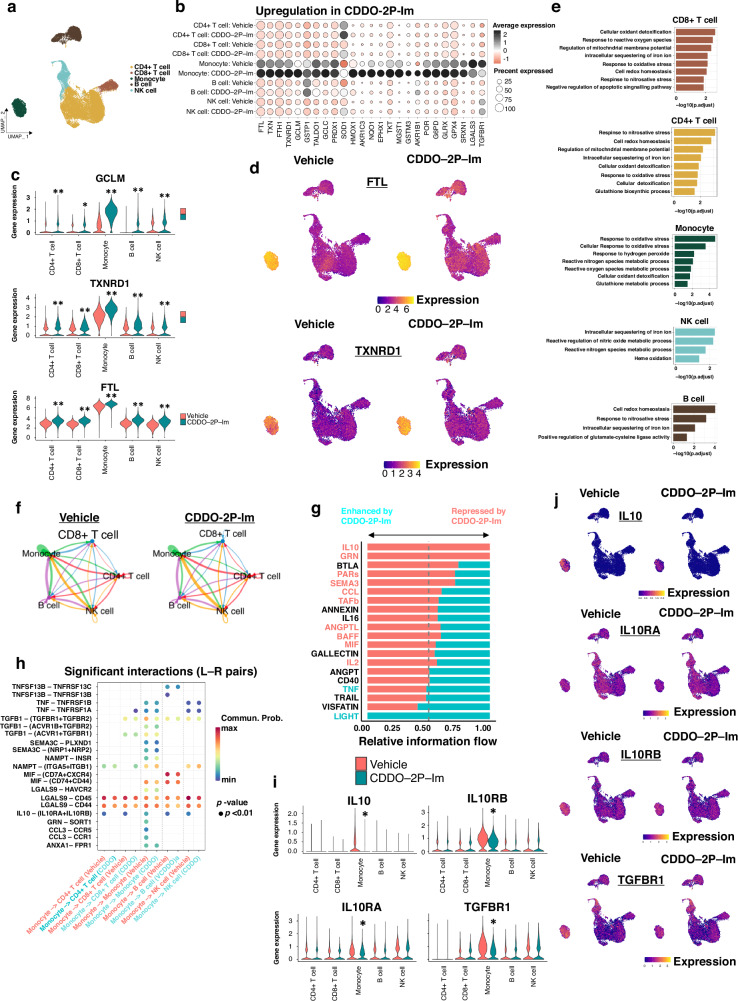
NRF2 induction broadly activates the anti-oxidative stress response, while repressing immunosuppressive IL10 and TGFβ signaling. **a** UMAP plot of the combined human PBMC scRNA-Seq data showing five distinct cell clusters, namely CD4+ T cells, CD8+ T cells, monocytes, B cells and NK cells. **b** Across all five PBMC clusters the electrophilic NRF2 activator CDDO-2P-Im induces broad and consistent upregulation of antioxidant and xenobiotic metabolism gene expression. **c** Violin plots showing that at the single cell level, the prototypical anti-oxidant pathway genes GCLM, TXNRD1 and FTL are significantly upregulated in all five clusters of immune cells from human PBMCs treated with 40 nM CDDO-2P-Im. **d** UMAP plots showing significant transcriptional upregulation of the prototypical anti-oxidant pathway genes FTL and TXNRD1 in all five clusters of immune cells from human PBMCs treated with 40 nM CDDO-2P-Im. **e** Pathway analysis of genes significantly upregulated by 40 nM CDDO-2P-Im shows consistent upregulation of pathways related to oxidative stress, redox homeostasis and oxidant detoxification across all five immune cell clusters in human PBMCs. **f** Overview of the cell-cell interactions in vehicle and CDDO-2P-Im-treated PBMCs across all five cell clusters as determined by CellChat. **g** Graphical representation of the relative information flow across different signaling pathways as determined by CellChat. Pathways which are repressed by CDDO-2P-Im treatment have extended red bars, whereas those activated by CDDO-2P-Im have extended blue bars. The names of the pathways highlighted in red are significantly repressed by CDDO-2P-Im, whereas those names highlighted in blue are significantly activated by CDDO-2P-Im treatment. **h** Ligand-receptor (L-R) pair interactions mediated by monocytes which were differentially regulated by CDDO-2P-Im treatment. Statistically significant interactions are denoted with a circle, and therefore, in the case of IL10 pathway interactions, the loss of the circle in response to CDDO-2P-Im treatment indicates repression of IL10 signaling across all cell types. **i** Violin plots showing that at the single cell level, the immunosuppressive genes IL10, IL10RA, IL10RB and TGFBR1 are all significantly downregulated specifically in the monocyte cluster of human PBMCs treated with 40 nM CDDO-2P-Im. **j** UMAP plots showing significant transcriptional downregulation of IL10, IL10RA, IL10RB and TGFBR1 genes specifically in the monocyte cluster of human PBMCs treated with 40 nM CDDO-2P-Im. *p < 0.005, **p < 0.0005.

Using the 10x Genomics scRNA-Seq platform, we analyzed a total of 40,681 cells from four human PBMC samples treated with either vehicle or CDDO-2P-Im, across two technical replicates (FIG S6A). Analysis of the data revealed five cell clusters: CD4+ T cells, CD8+ T cells, monocytes, B cells and NK cells (Fig. 3a, S6B–G, S7). NRF2 was expressed in all cell clusters (FIG S8), and thus all of the cell types analyzed have the potential to benefit from NRF2-induced cytoprotective gene expression.

Treatment of PBMCs with CDDO-2P-Im for 12 hrs had a significant impact on gene expression across all cell clusters, but did not significantly affect the cell type composition or cell cycle progression within each sample (FIG S9). Consistent with our expectations, NRF2 activation resulted in the significant and robust induction of anti-oxidative stress genes in all human donors across all cell clusters, exemplified by the genes *GCLM*, *TXNRD1* and *FTL*, which play a central role in the detoxification of reactive oxygen species (Fig. 3b–d, S10). In good agreement with this result, pathway level analysis revealed upregulation of “cellular oxidant detoxification”, “response to oxidative stress” and “cell redox homeostasis” across all five immune cell clusters (Fig. 3e). Taken together, these results support a model in which the treatment of immune effector cells with NRF2 activators will augment their anti-cancer functionality by alleviating the tumour microenvironment-induced oxidative stress which plays a significant role in repressing anti-cancer immunity.

### NRF2 inducers repress immunosuppressive IL10 and TGFβ signaling

As cell-cell communication plays a critical role in promoting immune cell effector functions, and NRF2 activation plays a significant role in the regulation of inflammatory gene expression [50, 63], we hypothesized that, in addition to regulating oxidative stress, NRF2 induction in immune cells may impact the cellular composition of the tumour through the modulation of cytokine and chemokine gene expression. Therefore, in order to determine the effects of NRF2 activation on immune cell-cell communication, we analysed our human PBMC scRNA-Seq data using CellChat [64] (Fig. 3f).

Analysis of information flow between cell types revealed that NRF2 inducer treatment has a significant impact on a number of important signaling pathways which play a critical role in regulating immune system functions (Fig. 3g). In this regard, we were particularly interested in the significant CDDO-2P-Im-dependent downregulation of IL10 and TGFβ signaling, as both of these pathways have immunosuppressive functions which may inhibit the anti-cancer immune response [26, 65]. Indeed, downregulation of IL10-mediated signaling from monocytes to other immune cell types was the most robust and broadly observed interaction amongst all signaling pathways analysed (Fig. 3g, S11). Specifically, IL10-mediated signaling from monocytes to CD4+ T cells, CD8+ T cells, monocytes, B cell and NK cells were all significantly repressed by CDDO-2P-Im, which argues that this immunosuppressive pathway is particularly responsive to NRF2 inducers (Fig. 3h).

Mechanistically, cell cluster-level gene expression analysis revealed that repression of IL10 signaling is mediated by both the transcriptional repression of the IL10 cytokine in monocytes, coupled with the repression of both subunits of the IL10 receptor (IL10RA and IL10RB) specifically in monocytes (Fig. 3i, j, S12A–C). The inhibition of IL10 gene expression and production by monocytes will inhibit the paracrine signaling to other immune effector cell types, while the repression of the IL10 receptor will further repress autocrine IL10-mediated signaling in monocytes. As IL10 predominantly functions as an immunosuppressive cytokine, through mechanisms which include repressing proinflammatory cytokine production [65], inhibition of IL10 signaling will function to stimulate immune cell activity.

In addition to IL10, CDDO-2P-Im treatment also resulted in repression of immunosuppressive TGFβ signaling in monocytes (Fig. 3i, j, S12D). This was caused by the monocyte-specific repression of the TGFBR1 receptor, which will function to reduce the signaling downstream of TGFβ ligand binding (Fig. 3i, j). Of note, as monocyte-derived cells can be broadly divided into two classes, anti-cancer M1 and pro-cancer M2 [28, 31], and both IL10 and TGFβ signaling are associated with the M2-lineage, these data suggest that NRF2 inducers function to promote myeloid anti-cancer immunity by repolarizing cells from the M2 to the anti-cancer M1 phenotype.

Taken together, these data show that, in addition to promoting anti-oxidative stress phenotypes, NRF2 inducers can also stimulate immune cell functions through the repression of immunosuppressive IL10 and TGFβ signaling pathways.

### NRF2 inducers repress the LGALS3-LAG3 immune checkpoint

**Fig. 4:**
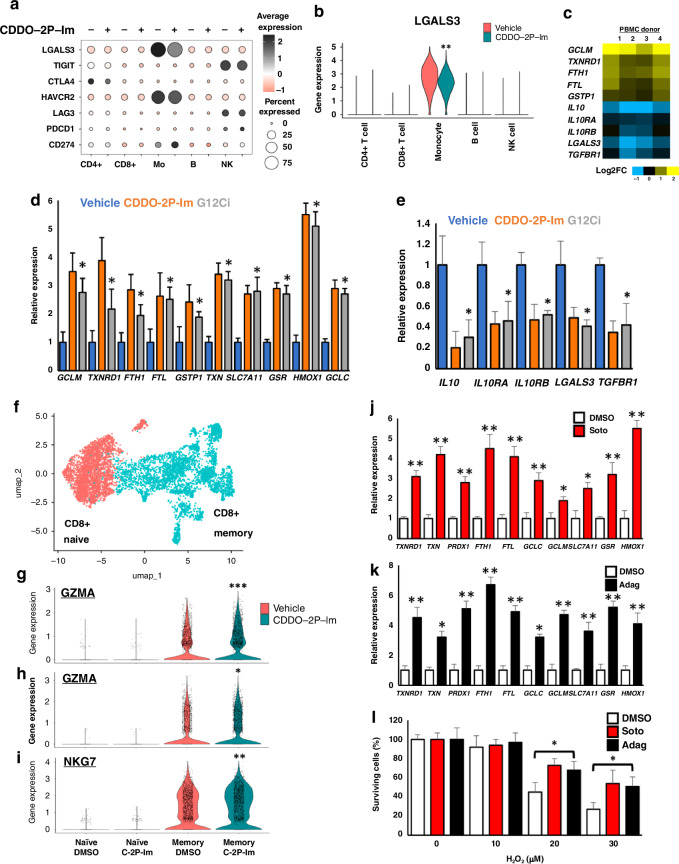
NRF2 inducers repress the LGALS3-LAG3 immune checkpoint. **a** Relative expression of immune checkpoint-related genes in human PBMCs treated with 40 nM CDDO-2P-Im, as determined by scRNA-Seq. Note that downregulation of LGALS3 in monocytes (Mo) is the only gene which is differentially regulated by CDDO-2P-Im treatment. **b** Violin plot showing that at the single cell level, the gene LGALS3, which is a ligand for the LAG-3 immune checkpoint, is significantly downregulated specifically in the monocyte cluster of human PBMCs treated with 40 nM CDDO-2P-Im. **c** Bulk RNA-Seq analysis showing the relative expression of a subset of anti-oxidative stress and immunosuppressive genes from human PBMCs treated with 40 nM CDDO-2P-Im. **d** The relative expression of prototypical anti-oxidative stress pathway genes in human PBMCs, treated with 40 nM CDDO-2P-Im, or 1 μM Adagrasib (G12Ci) for 12 h, determined using RT-qPCR. Data represent mean ± SD, *N* = 3. **e** The relative expression of immunosuppressive genes in human PBMCs, treated with 40 nM CDDO-2P-Im, or 1 μM Adagrasib (G12Ci) for 12 h, determined using RT-qPCR. **f** UMAP plot of the CD8+ T cell cluster from the human PBMC scRNA-Seq dataset. **g–i** Violin plots showing that at the single cell level, the CD8+ effector genes GZMA, GZMH and NKG7 are all significantly upregulated in CD8+ memory T cells after treatment with 40 nM CDDO-2P-Im. **j**, **k** The relative expression of prototypical anti-oxidative stress pathway genes in human proliferating T cells after treatment with 1 μM Sotorasib (Soto) or Adagrasib (Adag). **l** Pre-treatment with Sotorasib (red) or Adagrasib (black) increases cellular survival of human proliferating T cells in response to treatment with H_2_O_2_. Data represent mean ± SD, *N* = 3. *p < 0.05, **p < 0.005, *** *p* < 0.001.

In parallel to the immunomodulatory functions of cytokines, immune checkpoints constitute an additional mechanism through which anti-cancer immune surveillance is repressed within the tumour microenvironment [29]. To determine whether NRF2 inducers are also able to modulate immune cell functions through the transcriptional regulation of immune checkpoint genes, we analysed the gene expression profile of the PD1, CTLA4, TIGIT, LAG3 and TIM3 checkpoint pathways (Fig. 4a). Interestingly, while most immune checkpoint genes were unaffected by CDDO-2P-Im treatment, *LGALS3*, which encodes the galactin-3 protein, and functions as an activating ligand for the LAG3 T cell checkpoint [29], was significantly downregulated by CDDO-2P-Im treatment in monocytes (Fig. 4a, b). This downregulation of *LGALS3* in monocytes was observed in all 4 human PBMC samples, which demonstrates that the transcriptional repression of LGALS3 in human monocytes is a robust and reproducible effect (FIG S13). In this regard, the induction of anti-oxidative stress genes and repression of the IL10 pathway, *TGFBR1* and *LGALS3* genes were all reproduced in both bulk RNA-Seq, and RT-qPCR using an independent panel of 4 human PBMC samples (Fig. 4c–e).

In order to demonstrate the clinical relevance of these findings to NSCLC patients receiving electrophilic G12Ci anti-cancer drugs, we treated human PBMCs with Adagrasib (Fig. 4d, e). Consistent with our expectations, the RT-qPCR analysis revealed that the G12Ci was able to induce anti-oxidative stress genes, and repress *IL10*, *TGFBR1* and *LGALS3* gene expression in human PBMCs which did not contain a KRAS-G12C mutation, and thus was independent of KRAS inhibition.

As in mouse tumour models G12Ci treatment has been shown to directly augment CD8+ T cell effector functions, including by directly increasing granzyme gene expression [40], we were interested in exploring whether electrophilic inducers of NRF2 are able to promote a similar gene expression profile in human T cells. To this end, analysis of our scRNA-seq dataset revealed that in memory CD8+ T cells, CDDO-2P-Im treatment was able to significantly increase expression of the effector genes *GZMA*, *GZMH* and *NKG7* (Fig. 4f–i, S14). Together these data suggest that electrophilic activation of NRF2 may be in part responsible for augmented CD8+ T cell effector functions in response to G12Ci treatment.

Finally, due to nutrient limitations coupled with the chemically stressful nature of the tumour microenvironment, tumour infiltrating T cells are often subject to oxidative stress which can result in T cell dysfunction or programmed cell death [21–23]. As NRF2 activation promotes an anti-oxidative stress gene expression profile, we hypothesized that this in turn would increase T cell survival in response to oxidative stress. Consistent with our model, treatment of primary human proliferating T cells with either Sotorasib or Adagrasib broadly increased anti-oxidative stress gene expression (Fig. 4j, k) [66, 67]. Importantly, this gene expression program has phenotypic consequences, as it facilitates enhanced survival in response to hydrogen peroxide treatment (Fig. 4l), and therefore suggests that in the tumour niche, G12Ci treatment may improve anti-cancer immunity and T cell survival by the NRF2-dependent promotion of the anti-oxidative stress response.

**Fig. 5:**
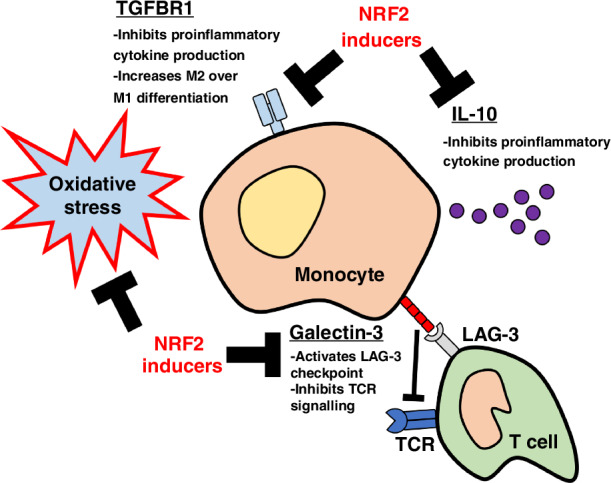
NRF2 promotes anti-cancer immunity through multiple parallel pathways. Activation of NRF2 in immune cells in the tumour microenvironment promotes anti-cancer immunity through multiple parallel pathways. In addition to inhibiting oxidative stress, which normally functions to repress T cell-mediated tumour immune surveillance, drug-induced activation of NRF2 in monocytes inhibits immunosuppressive IL10 and TGFβ signaling, while also repressing expression of the LAG3 checkpoint ligand galectin-3, which is encoded by the *LGALS3* gene.

In summary, our data suggest that, in addition to inhibiting the KRAS^G12C^ mutant protein, the clinically-approved KRAS inhibitors Sotorasib and Adagrasib are able to activate the NRF2-dependent anti-oxidative stress response through the direct electrophilic binding to KEAP1. As these drugs are administered orally, this will result in systemic activation of NRF2 throughout the cancer patients. In the immune system, the data support the model shown in Fig. 5, in which electrophilic NRF2 activators induce a unique gene expression profile in immune cells which functions to augment anti-cancer immunity through multiple parallel processes, and thus represents a novel immunotherapy which would complement existing immune checkpoint inhibitor drugs.

## Discussion

The development and clinical success of G12C mutant-specific KRAS inhibitors was a landmark achievement in anti-cancer drug development, as the oncogenic KRAS protein had long been considered an intractable therapeutic target [5, 6, 9]. Surprisingly, in this study we found that at physiologically-relevant doses, in addition to inhibiting KRAS, the clinically-approved KRAS^G12C^-inhibitors Sotorasib and Adagrasib also function as inducers of the transcription factor NRF2. Mechanistically, the same cysteine-targeting functionality which allows these electrophilic drugs to inhibit the mutant KRAS^G12C^ protein also facilitates binding to cysteine-based sensors in KEAP1, which results in NRF2 stabilization and the upregulation of NRF2-dependent gene expression. Importantly, this NRF2 activation in not dependent on the *KRAS*^*G12C*^ mutant status of the cells, and thus occurs independently of KRAS inhibition. In the malignant cells within the tumour, NRF2 activation will contribute to the efficacy of Sotorasib and Adagrasib through upregulation of NISP gene expression, which will promote immune cell infiltration into the tumour and anti-cancer immune surveillance. Concurrently, in the immune cells within the tumour microenvironment, electrophile-induced NRF2 activation improves resistance to oxidative stress, and will repolarize myeloid lineage cells towards the anti-cancer M1 sub-type. Thus, through the promotion of anti-cancer immunity through multiple parallel pathways, activation of NRF2 by Sotorasib and Adagrasib positively contributes to the clinical efficacy of this novel class of anti-cancer drugs.

The activation of NRF2 by G12Ci drugs provides a unique example of anti-cancer drugs which positively regulate the activity of a protein that is normally considered to be an oncogene. The therapeutic implication of this finding extends beyond NSCLC, as both Sotorasib and Adagrasib were recently included in the National Comprehensive Cancer Network guidelines as approved agents for PDAC tumours with *KRAS*^*G12C*^ mutations, while Adagrasib combined with Cetuximab was recently approved for the treatment of *KRAS*^*G12C*^ mutant colorectal cancer [3, 4]. Collectively, this means that cancer patients with lung, pancreatic and colorectal tumours will all be receiving an NRF2 inducer which will activate NRF2 gene expression throughout their entire bodies. In this regard, it is important to note that in human cancer, genetic activation of NRF2 follows a tissue specific pattern, whereby the NRF2 pathway is frequently activated in lung tumours, but much less so in colorectal and pancreatic tumours [2]. This mutation pattern presumably reflects the differential selective advantages which tumours of different cellular origins can gain from constitutive activation of NRF2, which in turn suggests that electrophile-mediated activation of NRF2 by G12C inhibitors may also differentially impact disease progression across the three tumour types.

In NSCLC, aberrant activation of NRF2 results in resistance to all current chemotherapeutics through the upregulation of detoxification enzymes and drug efflux transporters [32]. This extensively validated phenotype is highly relevant to *KRAS*^*G12C*^ mutant tumours due to ongoing efforts to increase the clinical effectiveness of G12Ci drugs. In this context, combination chemotherapy strategies using orthogonal cytotoxic agents are currently being pursued in order to increase the efficacy of G12Ci drugs, including co-administration of carboplatin (CodeBreaK 202), docetaxel (KRYSTAL-12) and the MEK inhibitor trametinib [3, 4]. In light of the fact that NRF2 activation provides direct resistance to platinum-based DNA damaging agents, microtubule poisons and trametinib [54], our current work suggests that these combination therapies may not provide the anticipated clinical benefit due to the electrophile-mediated NRF2 induction which forms an integral component of the cellular response to both Sotorasib and Adagrasib. As such, the fact that G12C inhibitors also induce NRF2 activity at clinical doses should be integrated into the logical reasoning and clinical decisions which are used to develop optimal drug combination strategies.

In a recent phase 3 clinical trial in which Sotorasib was co-administered with the EGFR inhibitor Panitumumab, the clinical response was significantly better for patients who received the higher dose of 960 mg, compared to the lower dose of 240 mg, per day of Sotorasib [68]. Pharmacokinetics studies have revealed that a 960 mg dose produces a blood plasma concentration of Sotorasib above 10 μM [46], which is more than 100-fold higher than the IC50 for KRAS inhibition [9], and consistent with the low micromolar concentrations required to induce NRF2 in this study. This clear clinical benefit provided by higher doses of Sotorasib strongly suggests that additional factors beyond the inhibition of KRAS contribute to the anti-cancer functionality of G12C inhibitors, of which activation of NRF2, induction of NISP gene expression, and the concomitant upregulation of anti-cancer immunity will play an important role. Consistent with this model, patients with *KEAP1* mutant tumours, which exhibit constitutive activation of NRF2 and therefore cannot benefit from G12Ci-induced NRF2 activity, have a poor prognosis in response to Sotorasib or Adagrasib [49, 56]. Thus, our model provides a mechanistic explanation for the clinical observation that *KEAP1* mutant NSCLC tumours respond poorly to G12Ci drugs, and argues that alternative treatment strategies would be more appropriate for patients who present with *KEAP1* or *NFE2L2* mutant NSCLC tumours.

The treatment of NSCLC has been revolutionized by the development of immune checkpoint inhibitor drugs targeting the PD-1/ PD-L1 pathway [69]. Unfortunately, only a subset of patients experience a clinical benefit from these drugs, with the efficacy and resistance often being determined by the tumour microenvironment-mediated repression of cytotoxic T cell functions. In this context, a broad range of experimental interventions, including the inhibition of oxidative stress, and repolarization of immunosuppressive macrophages, are being actively pursued in order to enhance effector T cell functions and augment the efficacy of immune checkpoint inhibitors. For example, the small molecule antioxidant N-acetylcysteine (NAC) restores cellular proliferation, increases cytokine production, and improves the killing capacity of chronically stimulated T cells [23]. Importantly, NAC treatment also synergizes with anti-PD-L1 therapy, which highlights the potential for targeting oxidative stress to enhance anti-cancer immunity. Similarly, inhibition of peroxinitrite, which is produced in a reaction between superoxide and nitric oxide, increases tumour infiltration of T cells and significantly increases the survival of tumour bearing mice [70]. Mechanistically, peroxinitrite produced by MDSCs directly inhibits tumour antigen recognition by T cells through the modification of the T cell receptor complex, which clearly highlights the central role that oxidative stress can play in inhibiting the anti-cancer immune response [71]. As drug-induced NRF2 activation in the tumour microenvironment will have a broad anti-oxidative effect across all immune cell types, we propose that small molecule modulation of NRF2 activity has the potential to function as a new complementary therapeutic strategy to enhance the anti-cancer immune response and synergize with existing immune checkpoint inhibitor treatment protocols.

As macrophages within the tumour microenvironment can also inhibit anti-cancer immunity and reduce the efficacy of immune checkpoint inhibitor drugs, therapeutic interventions which diminish this myeloid-based tumour promotion are the subject of intense research [28, 31]. For example, repression of PI3Kγ activity reprograms macrophages towards an anti-cancer phenotype through the upregulation of proinflammatory gene expression [30]. In this thematic context, we found that NRF2 activation represses immunosuppressive cytokine signaling mediated by IL-10 and TGFβ, which will function to repolarize myeloid-derived cells away from the tumour promoting M2-lineages, and therefore together, the available data support a model in which NRF2 inducers broadly reprogram the tumour microenvironment towards an anti-cancer phenotype by reversing the immunosuppressive functions of regulatory cells within the tumour niche.

In summary, in this study we found that, in addition to inhibiting oncogenic KRAS signaling, clinically-approved KRAS-G12C inhibitors also function as electrophilic inducers of NRF2, the activation of which promotes anti-cancer immunity through multiple parallel pathways, and thus positively contributes to the clinical efficacy of these anti-cancer drugs.

## Materials and methods

### Reagents

Sotorasib and ARS-1620 were purchased from Cayman Chemical (Ann Arbor, Michigan). Adagrasib and MRTX1133 were purchased from Selleck Chemicals (Texas). CDDO-2P-Im was a kind gift from Michael B. Sporn. For immunoblot analysis, anti-NRF2 (Santa Cruz, sc-13032) and anti-α-tubulin antibodies (Sigma, T9026) were used.

### Cell culture

All cancer cell lines were obtained from ATCC and were maintained in high glucose Dulbecco’s modified Eagle’s medium (DMEM), supplemented with 10% fetal bovine serum (FBS), and antibiotics. Frozen human PBMCs and proliferating T cells were obtained from ToMMo, and were thawed and re-suspended in RPMI 1640 medium supplemented with 10% FBS, and antibiotics. The PBMCs were seeded at a density of 400,000 cells per 35-mm dish, and after a 9-hour pre-culture, were treated with either 40 nM CDDO-2P-Im, or DMSO for 12 h. The proliferating T cells were activated using CD3/28 Dynabeads as previously described [66]. The NSCLC cell lines or proliferating human T cells were pre-treated with Sotorasib or Adagrasib for 48 hrs prior to treatment with either the chemotherapy drugs or hydrogen peroxide. All cells were cultured in a humidified atmosphere with 5% CO_2_ at 37 °C.

### Gene expression analysis

For RT-qPCR, total RNA was prepared from cell lysates using TRIzol reagent (Life Technologies, Carlsbad, California) in accordance with the manufacturer’s instructions. A 1 µg aliquot of total RNA was reverse transcribed with ReverTra Ace (Toyobo, Osaka). The resultant cDNA was used as a template for quantitative reverse transcription-PCR (qRT-PCR) on a SYBR green 7300 real time PCR analyzer (Life Technologies). The primers used during the qPCR analysis are available upon request. Gene set enrichment analysis of RNA-seq data from G12Ci-treated cells was carried out using the previously published GSE103021, GSE199582, GSE201412 and GSE224439 datasets. Analysis of NISP gene expression in *Pten:Keap1* DKO, *Atg7* CKO and Atg7:Keap1 DKO mice was carried out using the previously published GSE241215 and GSE50575 datasets.

### Surface Plasmon Resonance (SPR) analysis

Full-length His-tagged mouse Keap1 was immobilized on a Biacore CM5 sensor chip (Cytiva Life Sciences) using immobilization buffer (HBS-EP+, 1 mM TCEP). Sotorasib, Adagrasib, and Sulforaphane were diluted to 250 μM, and CDDO-Im was diluted to 100 μM, using running buffer (100 mM K/Na Phosphate, 100 mM NaCl, 1 mM GSH, 5% DMSO). These solutions were then injected into the flow cells at a flow rate of 10 μL/min for 120 s (association), followed by running buffer for 120 s (dissociation). Using a Biacore X100 the association phase of each sensorgram was analysed by plotting response unit (RU) values normalised to the molecular weight of the compounds.

### ELISA

The ELISA for CCL2 was carried out using the Mouse MCP1 ELISA Kit (ab208979, Abcam) following the protocol provided by the manufacturer. The samples were generated from conditioned media taken from WT and Keap1-Nrf2 DKO Hepa1 cells, which were treated with either DMSO or 10 μM Adagrasib (G12Ci) for 48 hrs.

### scRNA-Seq library preparation

After the 12 h treatment with CDDO-2P-Im or DMSO, all PBMCs were collected, which included use of Trypsin-EDTA (Nacalai Tesque, Kyoto) to remove attached cells, washed with PBS/0.04% BSA, and were re-suspended in PBS/0.04% BSA. After cell counting with Countess (Invitrogen), 10,000 cells were used for scRNA-Seq library preparation using the Chromium Next GEM Single Cell 3' Reagent Kits v3.1 Dual Index (10x GENOMICS). Sequencing libraries were then sequenced on the HiSeq 2500 (Illumina) and NovaSeq 6000 (Illumina) platforms to generate 28-bp and 90-bp reads.

### Bulk RNA-Seq library preparation

Total RNA was extracted from the PBMCs, including those attached to the dish, using the QIAGEN RNeasy Mini Kit (QIAGEN) according to the manufacturer's protocol. rRNA-depleted RNA-Seq libraries were prepared using the MGIEasy rRNA Depletion Kit (MGI) and MGIEasy RNA Directional Library Prep Set (MGI). Sequencing was performed on the DNBSEQ-G400 (MGI) platform to generate 150-bp paired-end reads.

### scRNA-Seq data analysis

The single-cell RNA sequencing (scRNA-seq) raw reads were aligned to the GRCh38 human reference using Cell Ranger (version 7.1.0) [72] provided by 10X Genomics. The unique molecular identifier (UMI) count matrices generated from Cell Ranger underwent cell selection based on the total number of detected genes and percentage of mitochondrial genes. Specifically, cells with fewer than 500 detected genes, more than 6,500 detected genes, and greater than 10% mitochondrial genes were considered low quality and thus discarded from further analysis. Genes that were expressed in fewer than three cells were filtered out from the final count matrices. To remove the potential doublets, DoubletFinder (version 2.0.3) [73] was used to analyze samples from four donors with two conditions (Vehicle and CDDO-2P-Im treatment) by setting the expected doublet rates as follows: 0.031 for donor 1, 0.04 for CDDO-2P-Im treatment in donor 2, 0.046 for Vehicle in donor 2, 0.05 for donor 3, and 0.039 for donor 4. A total of 40,681 cells from the eight samples were selected for the downstream analysis.

Seurat (version 4.9.9.9044) [74] was used for normalization, data scaling, dimensionality reduction, clustering, identifying differential expression, and most visualization. The UMI counts were normalized using NormalizeData function with the “LogNormalize” method and setting the scale factor to 10,000. Highly variable genes were identified by the FindVariableFeatures function with default parameters and used as input for principal components analysis by the RunPCA function after data scaling by the ScaleData function. Subsequently, batch correction and data integration were performed using Harmony (version 0.1.1) [75]. The first 30 principal components (PCs) were provided as an input for uniform manifold approximation and projection (UMAP) dimensionality reduction, constructing a shared nearest-neighbor graph, and unsupervised clustering analysis using RunUMAP, FindNeighbors, and FindClusters functions. The optimal resolution parameter used in the FindClusters function, which indirectly influences the number of clusters, was established based on the distinct signature expressions of each cell population and additional analyses including clustree (version 0.5.0) [76]. Next, cellular identity was determined by well-known and identified signatures for each cluster, and confirmed using SingleR (version 2.2.0) [77]. For identification of signatures of cell types, the FindAllMarkers function was used with the following parameters: Wilcoxon Rank Sum test, logfc.threshold = 1, and min.pct = 0.25. For analyzing differentially expressed genes (DEGs) between CDDO-2P-Im and Vehicle cells, the FindMarkers function was used with the following configurations: Wilcoxon Rank Sum test, logfc.threshold = 0.58, and min.pct = 0.25. Additionally, an adjusted p-value threshold of < 0.01 was applied to identify both signatures and DEGs.

CellChat (version 1.6.1) [64] was used to infer cell-cell communications (CCC) across various cell types and conditions. The Kyoto Encyclopedia of Genes and Genomes (KEGG) and Gene Ontology (GO) enrichment analysis, based on biological processes (BPs), was conducted using the clusterProfiler (version 4.8.2) [78]. The significant enrichment was filtered with a *p*-value of < 0.05. Cell cycle phases were analyzed with Seurat implemented CellCycleScoring function based on the expressions of 43 marker genes for the S-phase and 54 marker genes for the G2M-phase [79].

### Bulk RNA-Seq data analysis

The quality of the raw paired-end reads was initially assessed using FastQC (v0.11.9) (Babraham Bioinformatics, 2015). Subsequent quality control and trimming were performed with Fastp (v0.23.4), applying criteria including a minimum Phred score of 30, a minimum read length of 100 bp, and automatic adapter detection [80]. The trimmed reads were then aligned to the human reference genome GRCh38 using HISAT2 (v2.2.1) under default settings [81], resulting in SAM files which were subsequently converted to BAM format using samtools (v1.18) [82]. The transcripts of each sample were assembled and merged using StringTie (v2.1.4) [83]. The human gene annotation file (release 40) was sourced from GENCODE for accurate transcript annotation. A gene-level read count matrix was generated using the prepDE.py3 script from StringTie, facilitating downstream differential expression analysis. Differential expression between CDDO-2P-Im-treated and vehicle-treated samples was evaluated with DESeq2 (v1.42.1), incorporating adjustments for paired conditions [84]. Genes were deemed significantly differentially expressed at an adjusted p-value threshold of ≤ 0.05.

### Statistical analysis

For scRNA-Seq, the statistical differences were calculated by the Wilcoxon Rank Sum test. The analyses were performed using R (version 4.3.0). For RT-qPCR, the data are presented as mean ± SD. Student’s T-test (two-tailed) was used to determine the statistical significance of the results.

## Supplementary information

Supplementary Figures

## Data Availability

The human PBMC scRNA-Seq data have been deposited to the jMorp database and are available upon request (https://jmorp.megabank.tohoku.ac.jp).
