# Supplementary material for: Systemic activation of NRF2 contributes to the therapeutic efficacy of clinically-approved KRAS-G12C anti-cancer drugs
Source: Br J Cancer. 2025 Sep 1;133(9):1377–90. doi: 10.1038/s41416-025-03162-7 (PMC12572401; doi:10.1038/s41416-025-03162-7)
Supplement: Supplementary file 1 — Supplementary Figures [file 41416_2025_3162_MOESM1_ESM.pdf]

SUPPLEMENTARY FIGURES

Figure S1

The relative expression of representative classical NRF2 target genes in *KEAP1* mutant NSCLC cells treated with (**A, B**) 1 μM Sotorasib (Soto) or (**C**, **D**) 1 μM Adagrasib for 5 hrs, determined using RT-qPCR.

Figure S2

A. Chemical structure of ARS-1620. The cysteine-targeting electrophilic warhead is highlighted with a red star. **B.** The relative expression of representative classical NRF2 target genes in isogenic WT and Keap1-Nrf2 DKO Hepa1 cells after treatment 1 μM ARS-1620 for 5 hrs, determined using RT-qPCR. Each experiment was performed at least 3 times. Data represent mean ± SD, N = 6. **C-F.** The relative expression of representative classical NRF2 target genes in NSCLC cells treated with 1 μM ARS-1620 for 5 hrs, determined using RT-qPCR. A549 and H2023 have constitutive NRF2 activation due to inactivating mutations in *KEAP1*, whereas ABC1 and SW900 have a wild-type KEAP1-NRF2 pathway. Each experiment was performed at least 3 times. Data represent mean ± SD, N = 6, * $p < 0.01$, ns = not significant.

Figure S3

The molecular weight adjusted responses for the binding of electrophilic NRF2 activators to KEAP1 as determined using surface plasmon resonance (SPR).

Figure S4

A, B. Chemical structures of MRTX1257 and MRTX1133. Where present, the cysteine-targeting electrophilic warheads are highlighted with a red star. **C, D.** Enrichment plots for the "Xenobiotic Metabolism" hallmark gene set from the GSE103021 and GSE199582 datasets, which clearly shows significant induction of this gene signature by G12Ci treatment.

Figure S5

A. Mass spectrometry of Sotorasib-treated cells shows that the cysteine-288 sensor of KEAP1 can be bound by Sotorasib. Data were sourced from Canon et al. 2019. **B.** The same NISP genes that are induced in genetic mouse models of Nrf2 activation are also induced in an *in*

vivo mouse tumour model treated with Sotorasib (G12Ci). Mouse syngeneic *in vivo* tumour data were sourced from Canon et al. 2019, and gene expression analysis of *Pten:Keap1* DKO, *Atg7* CKO and Atg7:Keap1 DKO mice, all of which result in genetic activation of Nrf2, was carried out using the previously published GSE241215 and GSE50575 datasets.

Figure S6

A. Summary of the number of PBMCs analyzed by scRNA-Seq from each human donor and for each treatment condition. **B, C.** Combined totals and proportions of cells identified within the PBMC samples by scRNA-Seq across all five cell clusters. **D.** The top 5 gene signatures used to classify the PBMC scRNA-Seq data into the five cell clusters used throughout this study. **E, F, G.** Graphs showing the nCount_RNA, nFeature_RNA and percent.mt data for all five cell clusters identified from human PBMCs.

Figure S7

UMAP plots showing the gene expression patterns of some of the marker genes used to define the five cell clusters within the PBMC samples.

Figure S8

Violin plots from all four individual human PBMC donors showing that at the single cell level, the NFE2L2 gene, which encodes NRF2, is expressed in all five cellular clusters.

Figure S9

A. Table summarizing the total numbers of genes which were differentially regulated by CDDO-2P-Im treatment across all five cell clusters identified in human PBMCs. **B.** CDDO-2P-Im treatment does not impact the relative proportions of any of the five cell clusters identified in human PBMCs. **C.** CDDO-2P-Im treatment does not impact cell cycle progression in any of the five cell clusters identified in human PBMCs.

Figure S10

A-C. Violin plots from all four individual human PBMC donors showing that at the single cell level, the anti-oxidative stress genes GCLM (**A**), TXNRD1 (**B**) and FTL (**C**) are all significantly induced in all cell clusters in all donor PBMC samples treated with 40 nM CDDO-2P-Im.

Figure S11

Significant ligand-receptor (L-R) pair interactions between CD4+ T cells, CD8+ T cells, monocytes, B cells and NK cells which were differentially regulated by CDDO-2P-Im treatment. Statistically significant interactions are denoted with a circle, and therefore, in the case of IL10 pathway interactions, the loss of the circle in response to CDDO-2P-Im treatment indicates repression of IL10 signaling.

Figure S12

A-D. Violin plots from all four individual human PBMC donors showing that at the single cell level, the immunosuppressive genes IL10 (**A**), IL10RA (**B**), IL10RB (**C**) and TGFBR1 (**D**) are all significantly downregulated specifically in the monocyte cluster in all donor PBMC samples treated with 40 nM CDDO-2P-Im.

Figure S13

Violin plot from all four individual human PBMC donors showing that at the single cell level, the gene LGALS3, which is a ligand for the LAG-3 immune checkpoint, is significantly downregulated specifically in the monocyte cluster in all donor PBMC samples treated with 40 nM CDDO-2P-Im.

Figure S14

A,B. Representative UMAP plots for some of the markers used to define naïve and memory CD8+ T cells. **C-F.** Representative violin plots for some of the markers used to define naïve and memory CD8+ T cells. **G-I.** Representative violin plots demonstrating that anti-oxidative stress gene expression was significantly induced by CDDO-2P-Im treatment in both CD8+ T cell populations. * $p < 0.001$, ** $p < 0.001$.

Figure S1

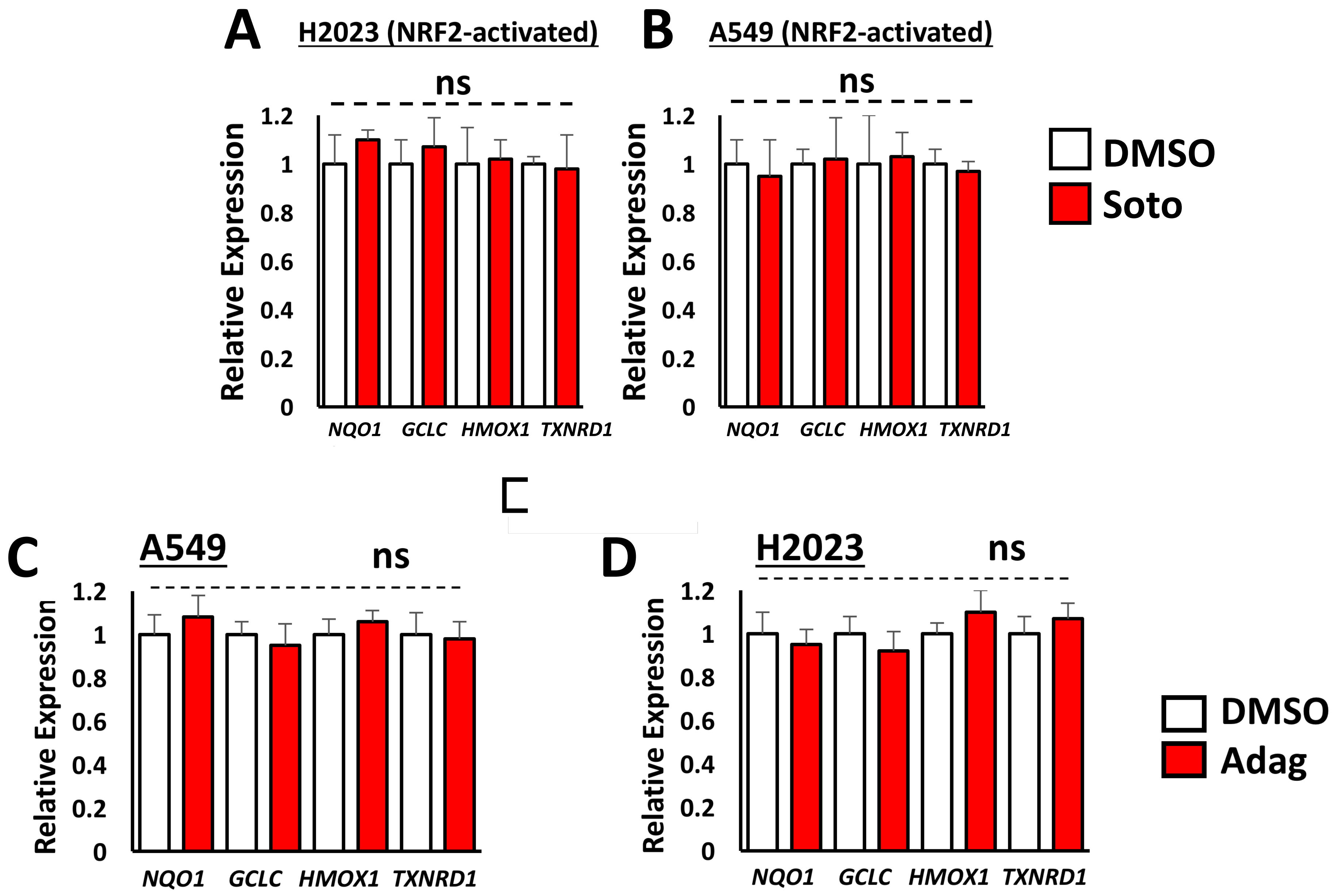

A
H2023 (NRF2-activated)
ns
B
A549 (NRF2-activated)
ns
Relative Expression
1.2
1
0.8
0.6
0.4
0.2
0
NQO1
GCLC
HMOX1
TXNRD1
DMSO
Soto
C
A549
ns
D
H2023
ns
DMSO
Adag

Figure S2

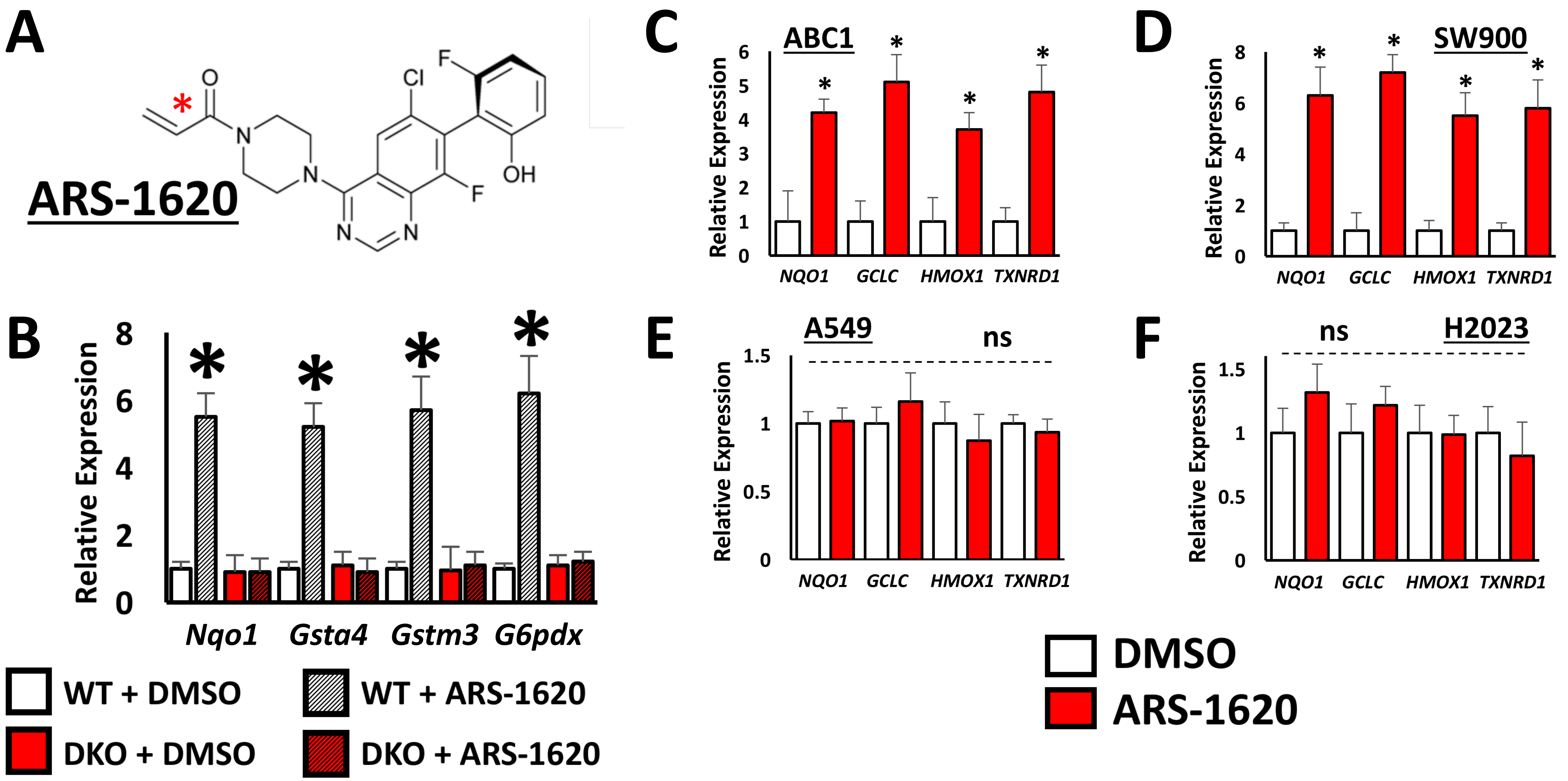
A
ARS-1620
O
Cl
F
N
N
OH
F
N
N
B
Relative Expression
8
6
4
2
0
Nqo1
Gsta4
Gstm3
G6pdx
WT + DMSO
WT + ARS-1620
DKO + DMSO
DKO + ARS-1620
C
ABC1
Relative Expression
6
5
4
3
2
1
0
NQO1
GCLC
HMOX1
TXNRD1
D
SW900
Relative Expression
8
6
4
2
0
NQO1
GCLC
HMOX1
TXNRD1
E
A549
ns
Relative Expression
1.5
1
0.5
0
NQO1
GCLC
HMOX1
TXNRD1
F
ns
H2023
Relative Expression
1.5
1
0.5
0
NQO1
GCLC
HMOX1
TXNRD1
DMSO
ARS-1620

Figure S3

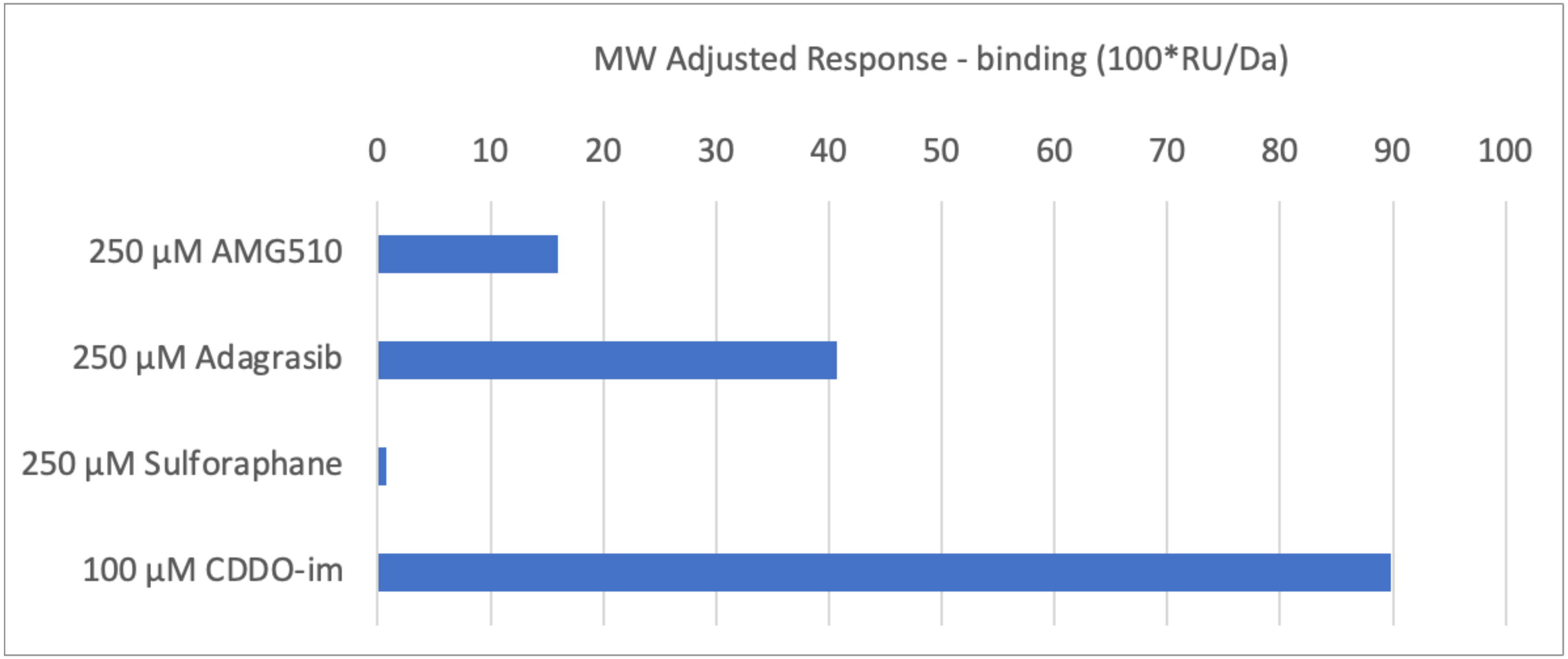

20250423 Keap1-compounds	immobilize	12983.2 RU
	MW	MW adjusted response (100*RU/Da)
250 µM AMG510	560.61	15.90979385
250 µM Adagrasib	604.12	40.67612076
250 µM Sulforaphane	177.29	0.716810524
100 µM CDDO-im	541.72	89.76150513

Figure S4

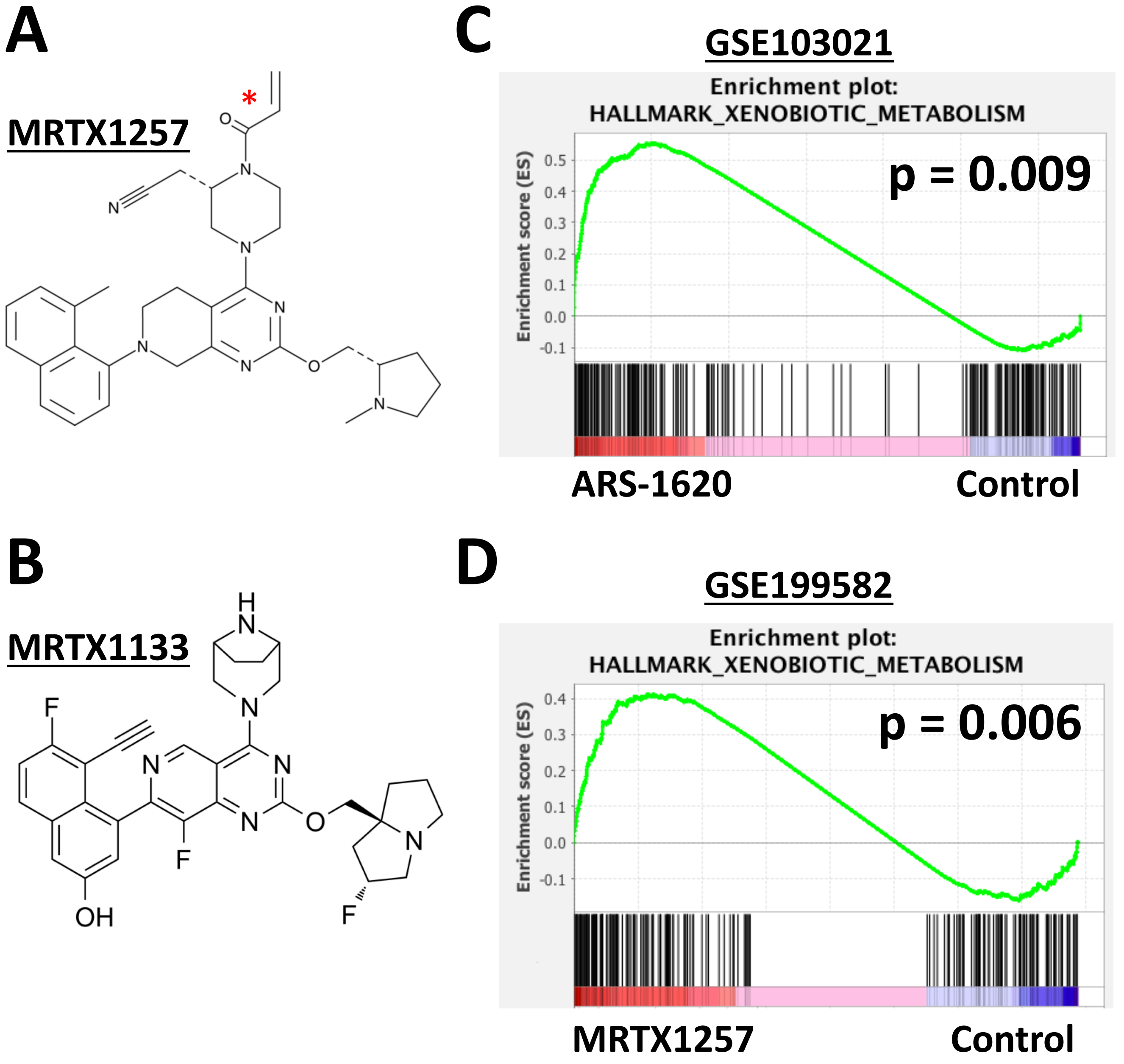
A
MRTX1257
B
MRTX1133
C
GSE103021
Enrichment plot:
HALLMARK_XENOBIOTIC_METABOLISM
Enrichment score (ES)
p = 0.009
ARS-1620
Control
D
GSE199582
Enrichment plot:
HALLMARK_XENOBIOTIC_METABOLISM
Enrichment score (ES)
p = 0.006
MRTX1257
Control

Figure S5

A

Protein ID	Gene Symbol	Peptide Sequence
P01116-G2C	KRAS	K.LVVVGAC#GVGK.S = Cys12
Q14145	KEAP1	K.C#EILQSDSR.C = Cys288

B

Chemokine/ Receptor	Mouse model			
	Syngeneic tumour + G12Ci	*Pten: Keap1* DKO	*Atg7* CKO	*Atg7:Keap1* DKO
	Pathway score p-value	Fold Induction		
CCL2	7.33E-06	46.37	16.45	15.98
CCL7	5.31E-05	4.31	16.64	12.89
CXCL5	1.02E-03	117.61	13.59	15.06
CXCL14	1.05E-04	8.46	2.55	2.3
CX3CL1	3.29E-02	11.98	11.64	9.67
CCR2	7.34E-07	11.46	12.72	27.5
CX3CR1	2.74E-06	8.48	66.55	55.84

Figure S6

A

	Treatment	Cell Number
Donor 1	Vehicle	3,730
	CDDO-2P-Im	3,998
Donor 2	Vehicle	5,623
	CDDO-2P-Im	5,011
Donor 3	Vehicle	6,272
	CDDO-2P-Im	6,287
Donor 4	Vehicle	4,806
	CDDO-2P-Im	4,954
TOTAL		40,681

B

	Cell Number
CD4+ T cell	23,747
CD8+ T cell	3,280
Monocyte	4,491
B cell	5,178
NK cell	3,985
TOTAL	40,681

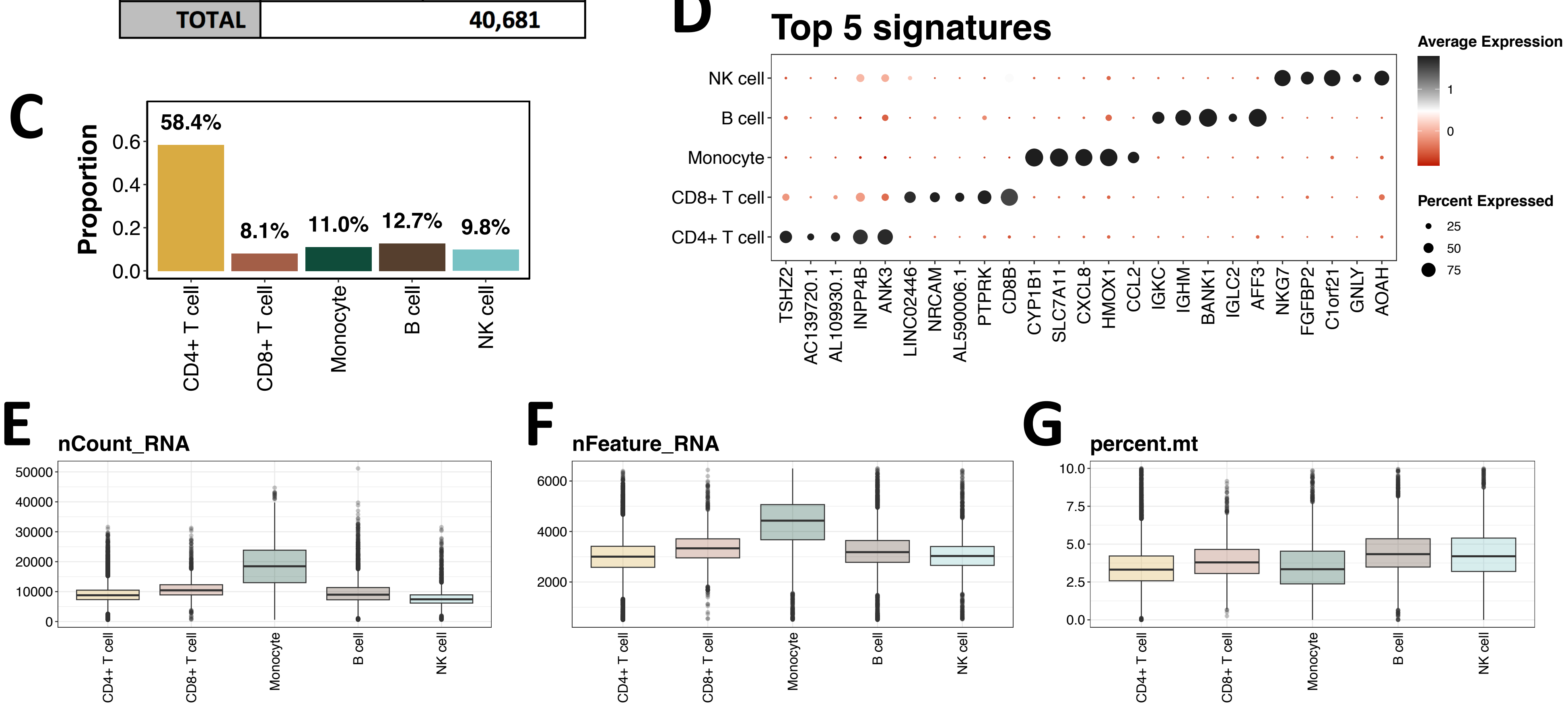

Figure S7

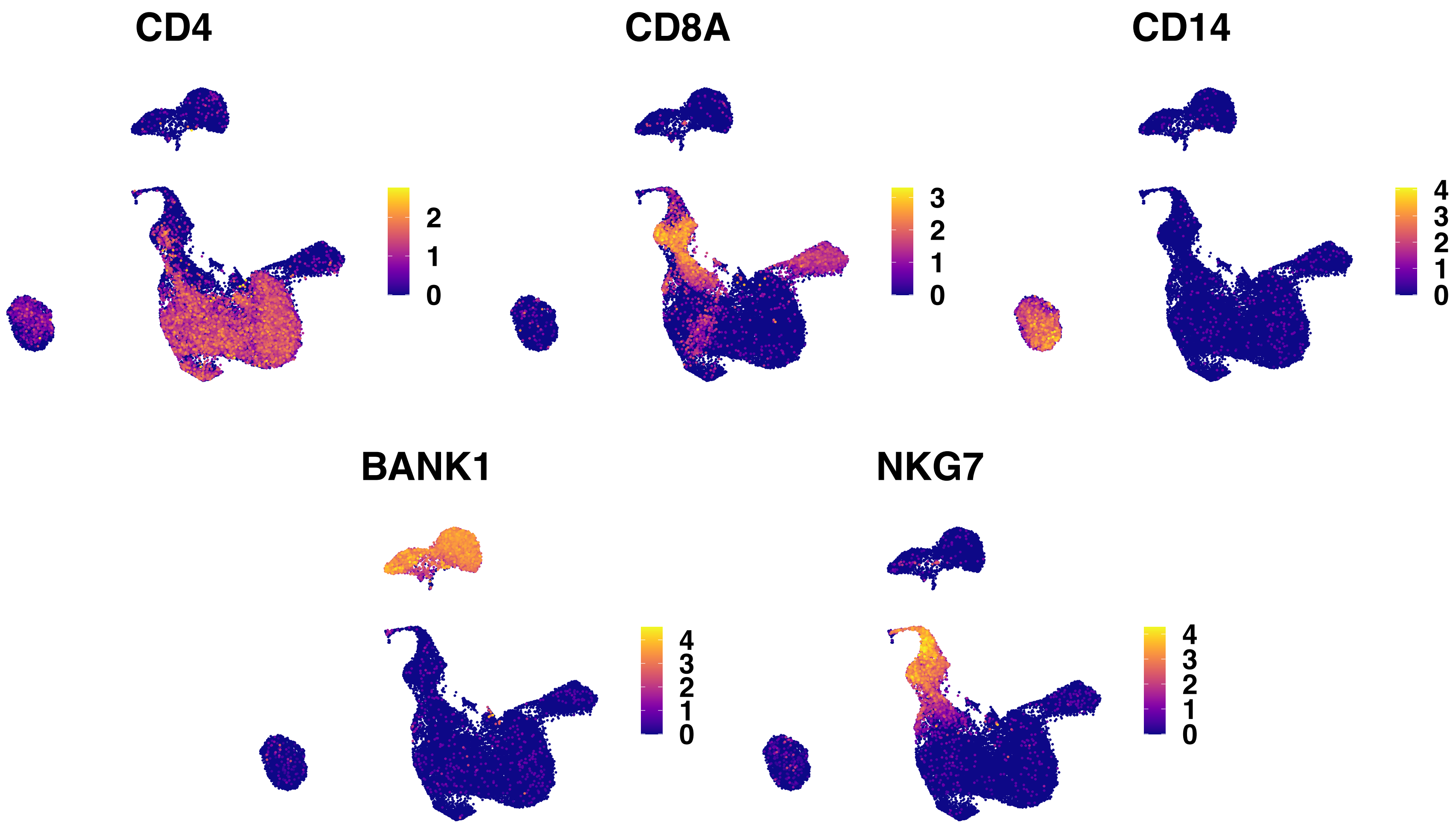

Figure S8

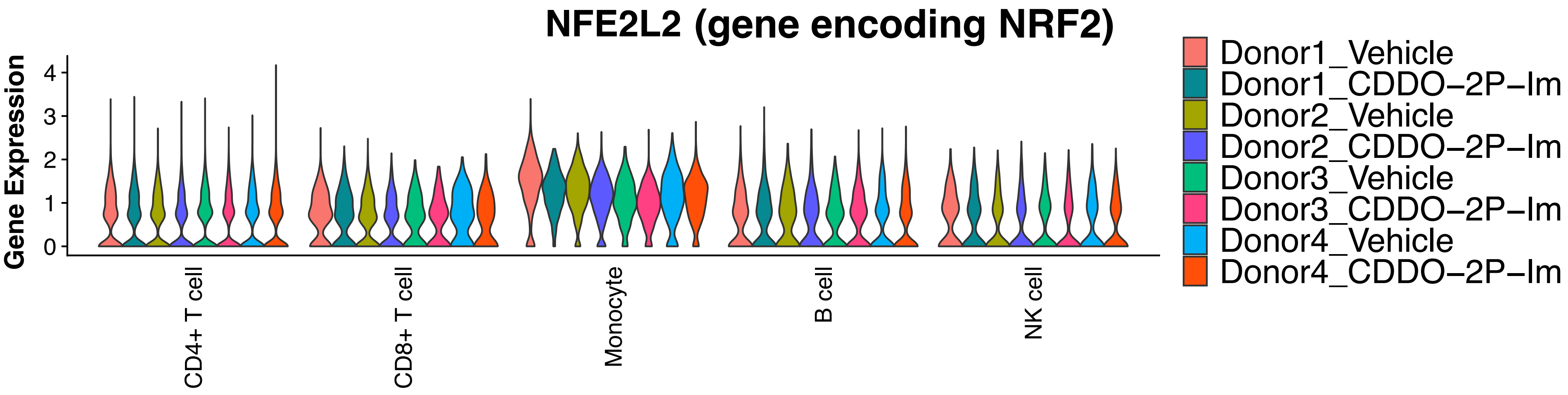
NFE2L2 (gene encoding NRF2)
Gene Expression
0
1
2
3
4
CD4+ T cell
CD8+ T cell
Monocyte
B cell
NK cell
Donor1_Vehicle
Donor1_CDDO−2P−Im
Donor2_Vehicle
Donor2_CDDO−2P−Im
Donor3_Vehicle
Donor3_CDDO−2P−Im
Donor4_Vehicle
Donor4_CDDO−2P−Im

Figure S9

A

	Differentially Expressed Genes	
	Upregulated	Downregulated
CD4+ T cell	66	62
CD8+ T cell	51	42
Monocyte	555	878
B cell	89	88
NK cell	43	70

B

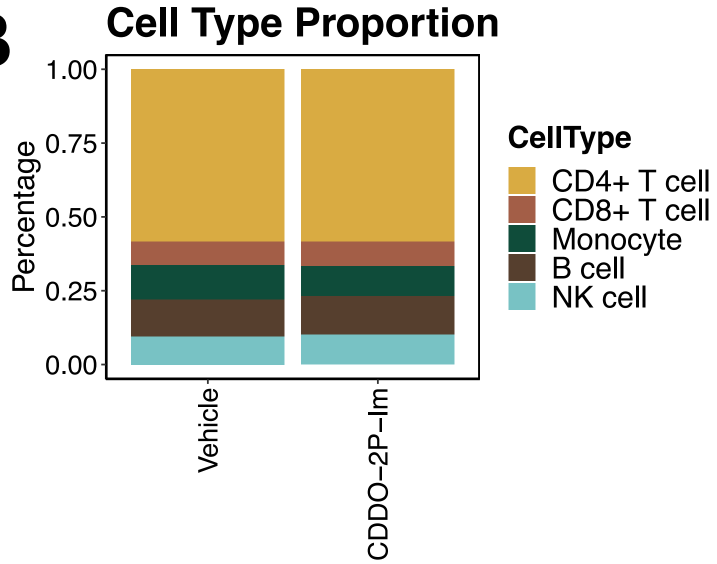

C

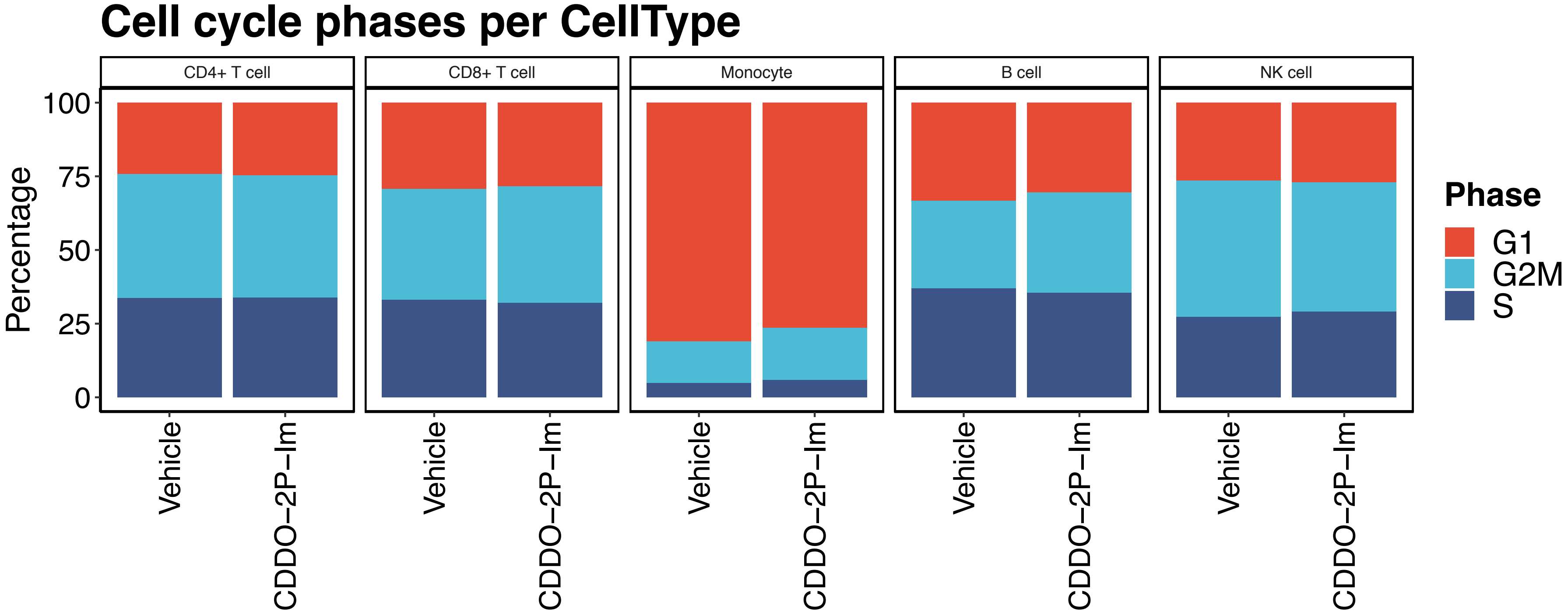

Figure S10

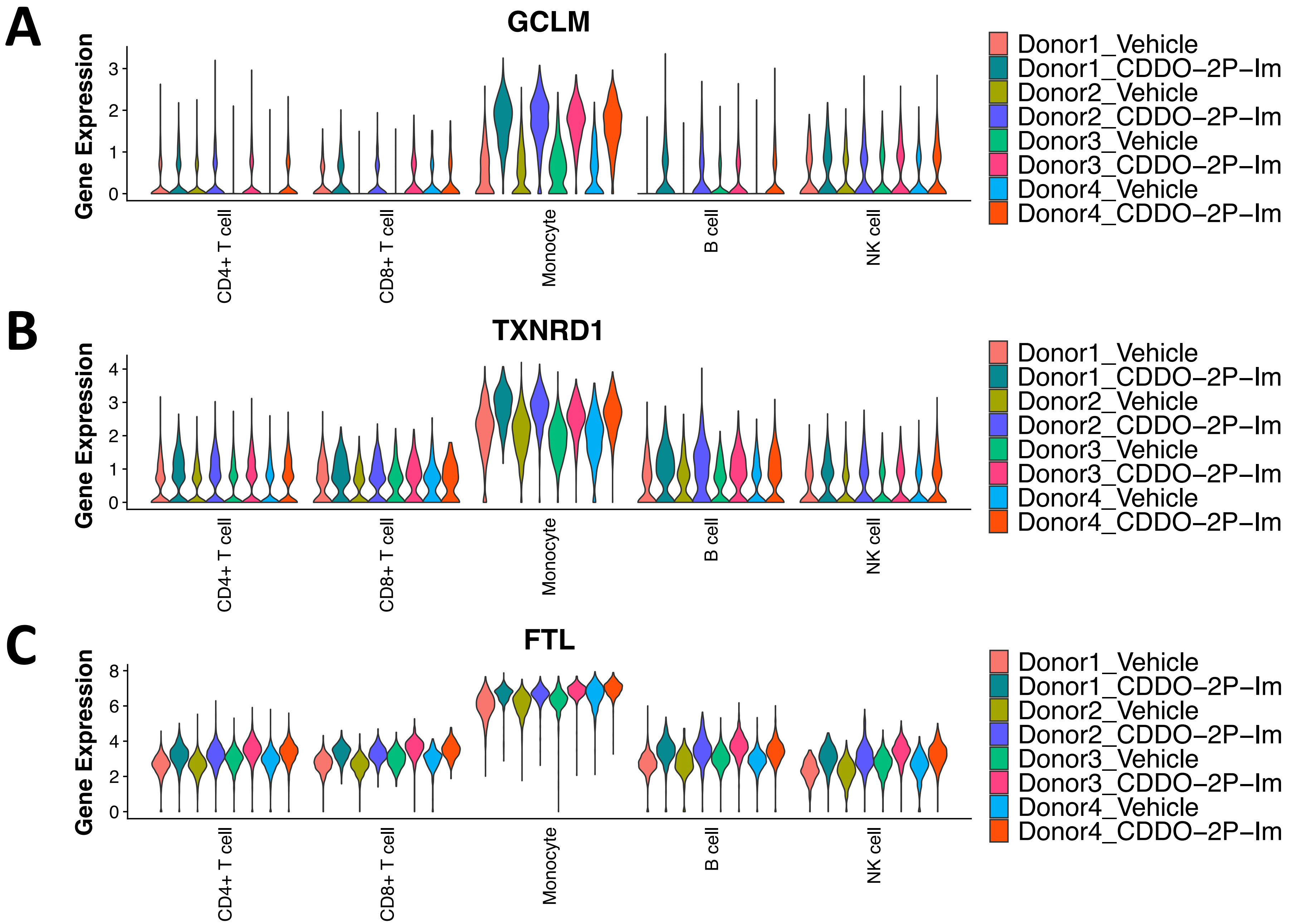
A
GCLM
Gene Expression
0
1
2
3
CD4+ T cell
CD8+ T cell
Monocyte
B cell
NK cell
Donor1_Vehicle
Donor1_CDDO−2P−Im
Donor2_Vehicle
Donor2_CDDO−2P−Im
Donor3_Vehicle
Donor3_CDDO−2P−Im
Donor4_Vehicle
Donor4_CDDO−2P−Im
B
TXNRD1
Gene Expression
0
1
2
3
4
CD4+ T cell
CD8+ T cell
Monocyte
B cell
NK cell
Donor1_Vehicle
Donor1_CDDO−2P−Im
Donor2_Vehicle
Donor2_CDDO−2P−Im
Donor3_Vehicle
Donor3_CDDO−2P−Im
Donor4_Vehicle
Donor4_CDDO−2P−Im
C
FTL
Gene Expression
0
2
4
6
8
CD4+ T cell
CD8+ T cell
Monocyte
B cell
NK cell
Donor1_Vehicle
Donor1_CDDO−2P−Im
Donor2_Vehicle
Donor2_CDDO−2P−Im
Donor3_Vehicle
Donor3_CDDO−2P−Im
Donor4_Vehicle
Donor4_CDDO−2P−Im

Figure S11

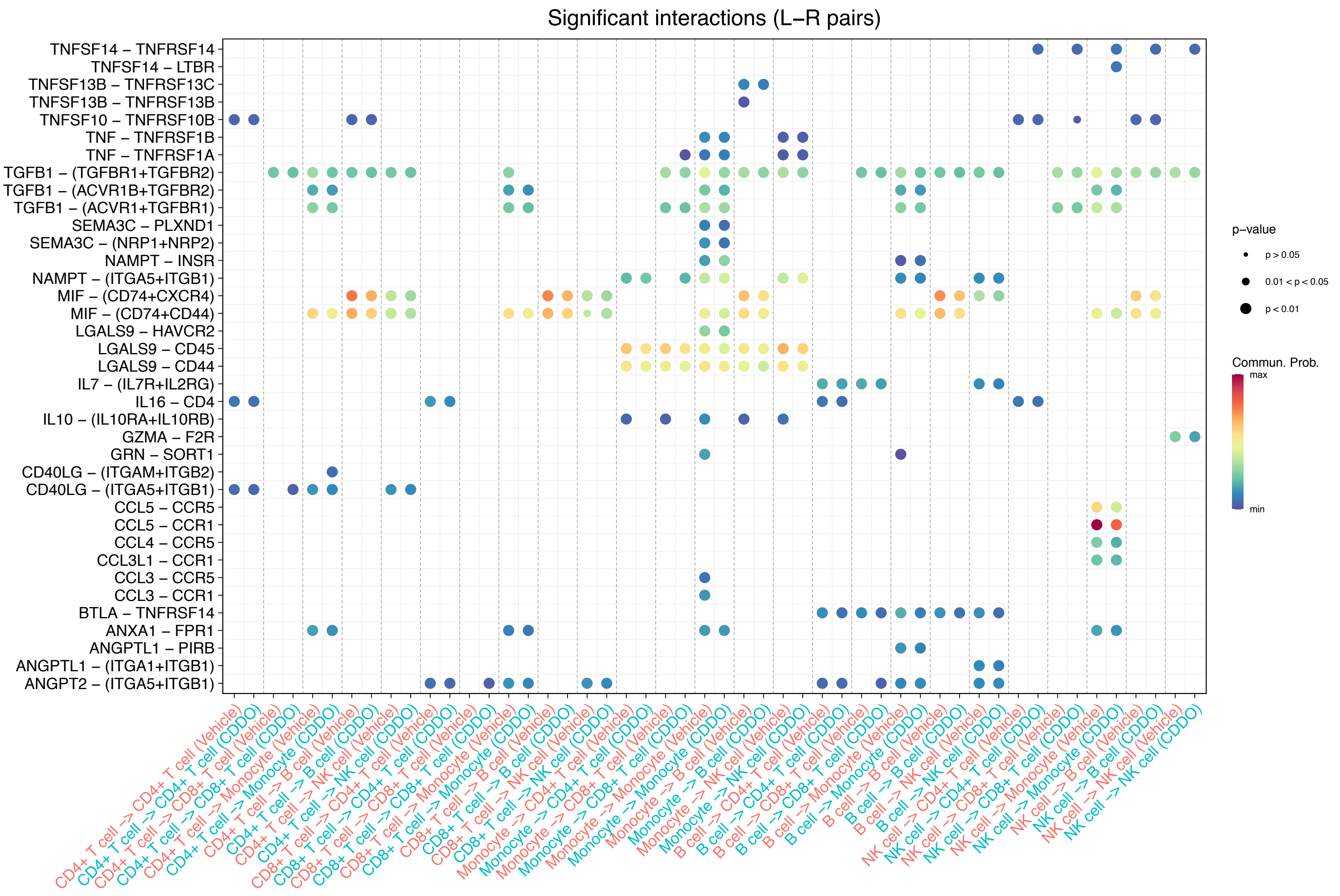

Significant interactions (L–R pairs)
TNFSF14 – TNFRSF14
TNFSF14 – LTBR
TNFSF13B – TNFRSF13C
TNFSF13B – TNFRSF13B
TNFSF10 – TNFRSF10B
TNF – TNFRSF1B
TNF – TNFRSF1A
TGFB1 – (TGFBR1+TGFBR2)
TGFB1 – (ACVR1B+TGFBR2)
TGFB1 – (ACVR1+TGFBR1)
SEMA3C – PLXND1
SEMA3C – (NRP1+NRP2)
NAMPT – INSR
NAMPT – (ITGA5+ITGB1)
MIF – (CD74+CXCR4)
MIF – (CD74+CD44)
LGALS9 – HAVCR2
LGALS9 – CD45
LGALS9 – CD44
IL7 – (IL7R+IL2RG)
IL16 – CD4
IL10 – (IL10RA+IL10RB)
GZMA – F2R
GRN – SORT1
CD40LG – (ITGAM+ITGB2)
CD40LG – (ITGA5+ITGB1)
CCL5 – CCR5
CCL5 – CCR1
CCL4 – CCR5
CCL3L1 – CCR1
CCL3 – CCR5
CCL3 – CCR1
BTLA – TNFRSF14
ANXA1 – FPR1
ANGPTL1 – PIRB
ANGPTL1 – (ITGA1+ITGB1)
ANGPT2 – (ITGA5+ITGB1)
CD4+ T cell -> CD4+ T cell (Vehicle)
CD4+ T cell -> CD4+ T cell (CDDO)
CD4+ T cell -> CD8+ T cell (Vehicle)
CD4+ T cell -> CD8+ T cell (CDDO)
CD4+ T cell -> Monocyte (Vehicle)
CD4+ T cell -> Monocyte (CDDO)
CD4+ T cell -> B cell (Vehicle)
CD4+ T cell -> B cell (CDDO)
CD4+ T cell -> NK cell (Vehicle)
CD4+ T cell -> NK cell (CDDO)
CD8+ T cell -> CD4+ T cell (Vehicle)
CD8+ T cell -> CD4+ T cell (CDDO)
CD8+ T cell -> CD8+ T cell (Vehicle)
CD8+ T cell -> CD8+ T cell (CDDO)
CD8+ T cell -> Monocyte (Vehicle)
CD8+ T cell -> Monocyte (CDDO)
CD8+ T cell -> B cell (Vehicle)
CD8+ T cell -> B cell (CDDO)
CD8+ T cell -> NK cell (Vehicle)
CD8+ T cell -> NK cell (CDDO)
Monocyte -> CD4+ T cell (Vehicle)
Monocyte -> CD4+ T cell (CDDO)
Monocyte -> CD8+ T cell (Vehicle)
Monocyte -> CD8+ T cell (CDDO)
Monocyte -> Monocyte (Vehicle)
Monocyte -> Monocyte (CDDO)
Monocyte -> B cell (Vehicle)
Monocyte -> B cell (CDDO)
Monocyte -> NK cell (Vehicle)
Monocyte -> NK cell (CDDO)
B cell -> CD4+ T cell (Vehicle)
B cell -> CD4+ T cell (CDDO)
B cell -> CD8+ T cell (Vehicle)
B cell -> CD8+ T cell (CDDO)
B cell -> Monocyte (Vehicle)
B cell -> Monocyte (CDDO)
B cell -> B cell (Vehicle)
B cell -> B cell (CDDO)
B cell -> NK cell (Vehicle)
B cell -> NK cell (CDDO)
NK cell -> CD4+ T cell (Vehicle)
NK cell -> CD4+ T cell (CDDO)
NK cell -> CD8+ T cell (Vehicle)
NK cell -> CD8+ T cell (CDDO)
NK cell -> Monocyte (Vehicle)
NK cell -> Monocyte (CDDO)
NK cell -> B cell (Vehicle)
NK cell -> B cell (CDDO)
NK cell -> NK cell (Vehicle)
NK cell -> NK cell (CDDO)
p–value
p > 0.05
0.01 < p < 0.05
p < 0.01
Commun. Prob.
max
min

Figure S12

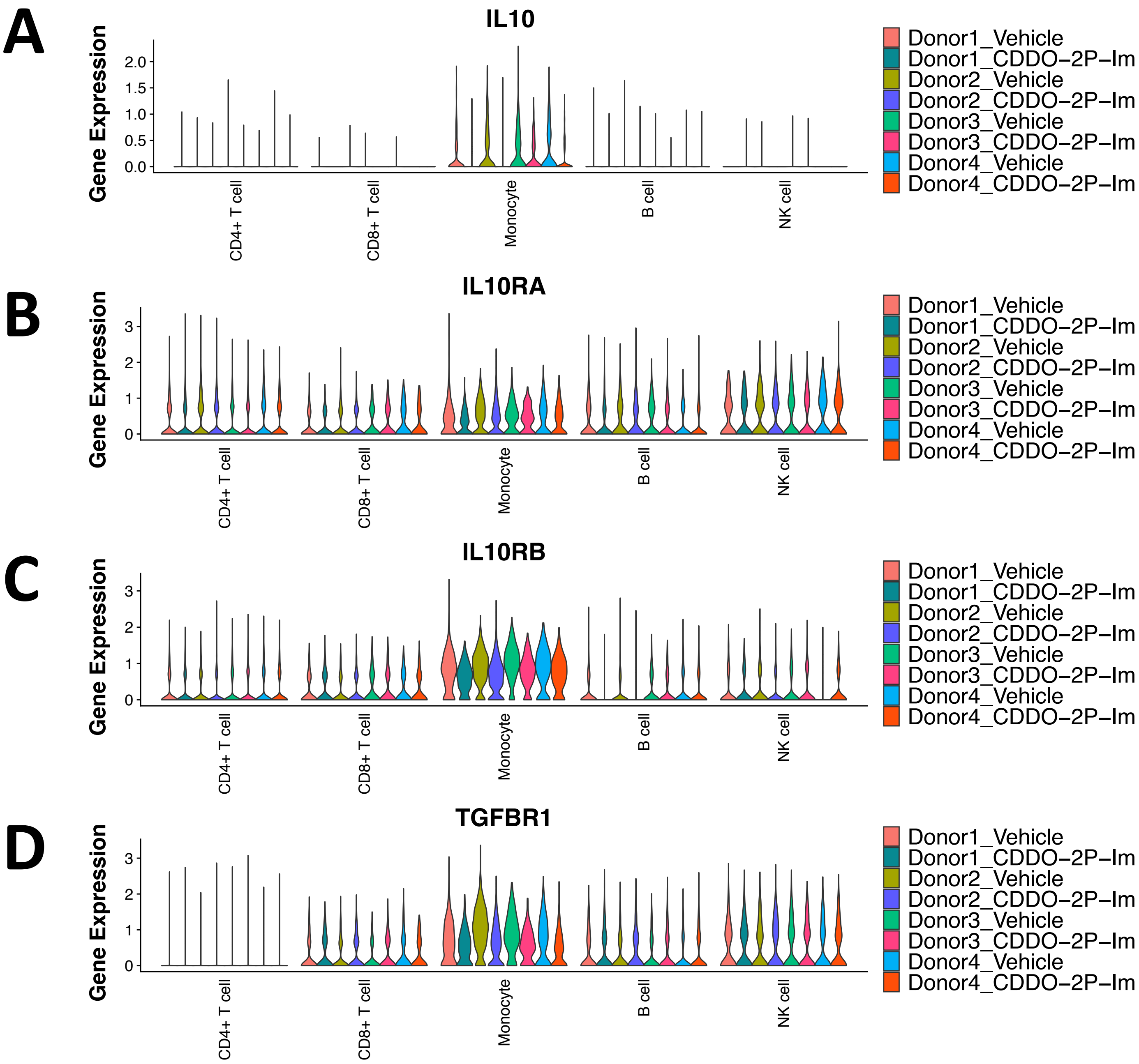

A
IL10
Gene Expression
2.0
1.5
1.0
0.5
0.0
CD4+ T cell
CD8+ T cell
Monocyte
B cell
NK cell
Donor1_Vehicle
Donor1_CDDO−2P−Im
Donor2_Vehicle
Donor2_CDDO−2P−Im
Donor3_Vehicle
Donor3_CDDO−2P−Im
Donor4_Vehicle
Donor4_CDDO−2P−Im
B
IL10RA
Gene Expression
3
2
1
0
CD4+ T cell
CD8+ T cell
Monocyte
B cell
NK cell
Donor1_Vehicle
Donor1_CDDO−2P−Im
Donor2_Vehicle
Donor2_CDDO−2P−Im
Donor3_Vehicle
Donor3_CDDO−2P−Im
Donor4_Vehicle
Donor4_CDDO−2P−Im
C
IL10RB
Gene Expression
3
2
1
0
CD4+ T cell
CD8+ T cell
Monocyte
B cell
NK cell
Donor1_Vehicle
Donor1_CDDO−2P−Im
Donor2_Vehicle
Donor2_CDDO−2P−Im
Donor3_Vehicle
Donor3_CDDO−2P−Im
Donor4_Vehicle
Donor4_CDDO−2P−Im
D
TGFBR1
Gene Expression
3
2
1
0
CD4+ T cell
CD8+ T cell
Monocyte
B cell
NK cell
Donor1_Vehicle
Donor1_CDDO−2P−Im
Donor2_Vehicle
Donor2_CDDO−2P−Im
Donor3_Vehicle
Donor3_CDDO−2P−Im
Donor4_Vehicle
Donor4_CDDO−2P−Im

Figure S13

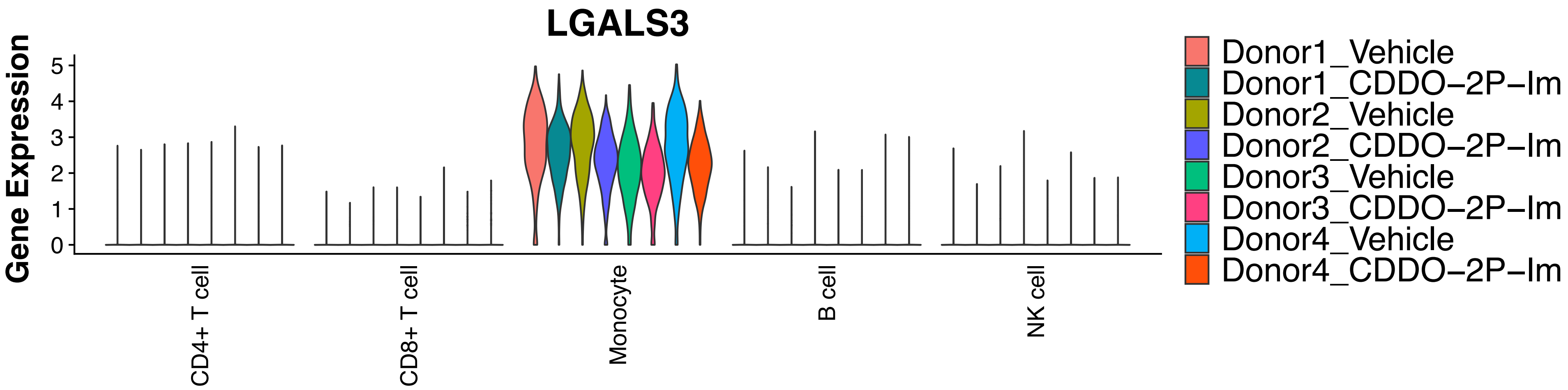
LGALS3
Gene Expression
0
1
2
3
4
5
CD4+ T cell
CD8+ T cell
Monocyte
B cell
NK cell
Donor1_Vehicle
Donor1_CDDO−2P−Im
Donor2_Vehicle
Donor2_CDDO−2P−Im
Donor3_Vehicle
Donor3_CDDO−2P−Im
Donor4_Vehicle
Donor4_CDDO−2P−Im

Figure S14

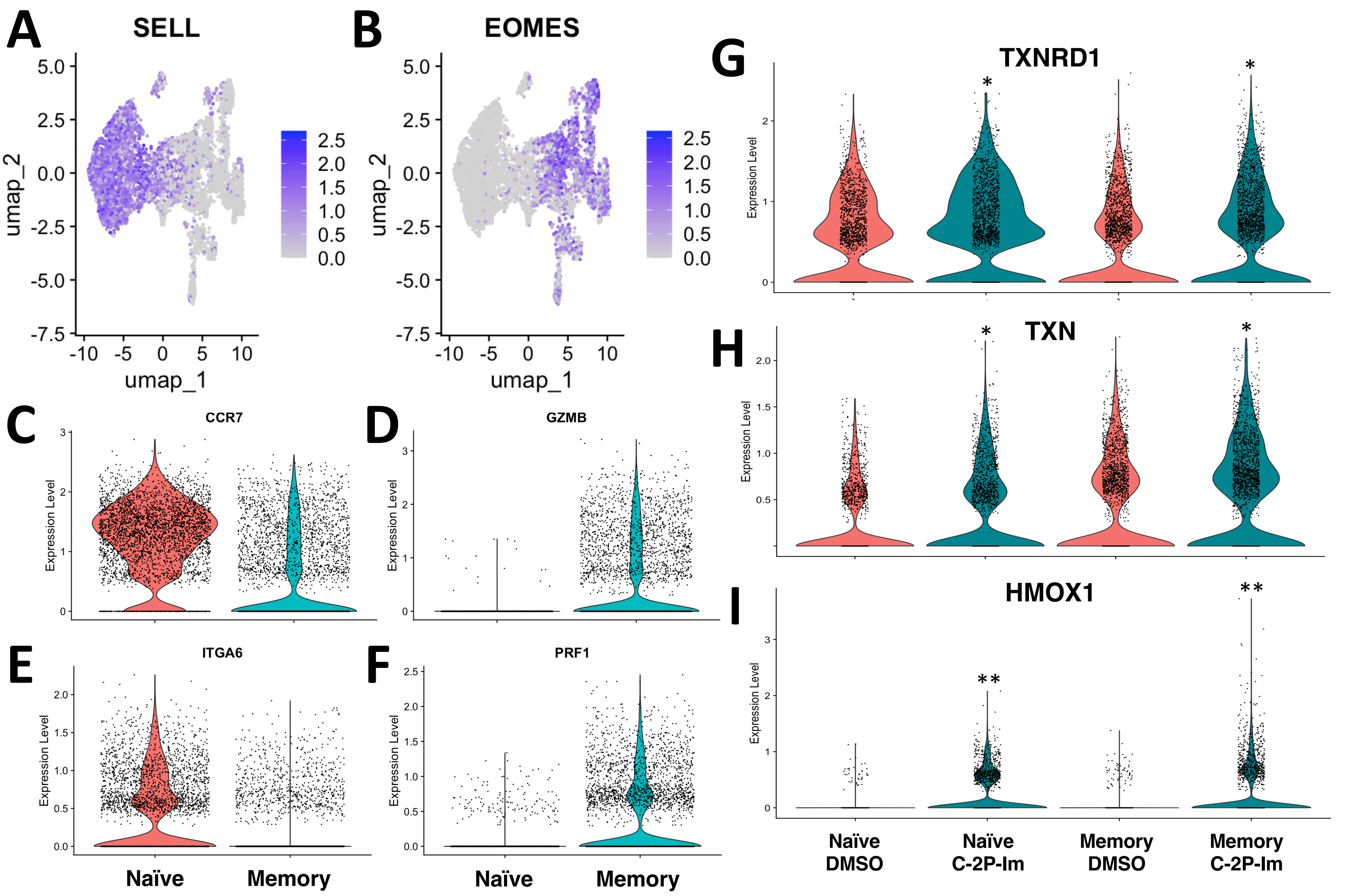

A
SELL
B
EOMES
umap_1
umap_2
C
CCR7
D
GZMB
E
ITGA6
F
PRF1
Expression Level
Naïve
Memory
G
TXNRD1
H
TXN
I
HMOX1
Naïve DMSO
Naïve C-2P-Im
Memory DMSO
Memory C-2P-Im
